# MIKC-type MADS-box transcription factor OsMADS31 positively regulates salinity tolerance in rice

**DOI:** 10.3389/fpls.2025.1628305

**Published:** 2025-09-04

**Authors:** Xiaolin Yin, Qinmei Gao, Feng Wang, Weihao Liu, Shixuan Yu, Shuixiu Zhong, Jiahui Feng, Rui Bai, Yiting Luo, Liangbi Chen, Xiaojun Dai, Manzhong Liang

**Affiliations:** Hunan Province Key Laboratory of Crop Sterile Germplasm Resource Innovation and Application, College of Life Science, Hunan Normal University, Changsha, China

**Keywords:** rice (*Oryza sativa* L.), MADS-box, transcription factor, OsMADS31, salt stress, antioxidant, transcriptome

## Abstract

Rice (*Oryza sativa* L.) is one of the world’s most vital staple crops, providing food for over 50% of the global population. As a salt-sensitive crop, rice is susceptible to damage from soil-soluble salt stress, which can severely reduce rice yield. Here, we aimed to elucidate the molecular mechanisms underlying salt tolerance in rice. Investigation of MADS-box genes involved in abiotic stress responses in rice led to the identification of *OsMADS31*. To investigate the role of *OsMADS31* in salt stress tolerance, we generated its knockout mutant and overexpression lines in Nipponbare (Nip). Phenotypic analysis of T_2_-generation *OsMADS31* knockout (*osmads31*) mutants revealed altered panicle morphology and significant reductions in seed-setting rate, panicle length, grain number per panicle, and 1000-grain weight. Under salt stress, both during seed germination and at the three-leaf stage, *osmads31* knockout mutants exhibited markedly inhibited growth, whereas *OsMADS31* overexpression (*OE*) lines maintained normal germination and development. At the three-leaf stage, knockout mutants showed significantly lower survival rates following salt treatment and subsequent recovery. Physiological and biochemical assays demonstrated that, compared with wild-type (WT) plants, *osmads31* mutants exhibited substantially decreased catalase (CAT), peroxidase (POD), and superoxide dismutase (SOD) activities as well as reduced proline (Pro) content. Conversely, compared with WT plants, 3,3’-diaminobenzidine (DAB) and nitroblue tetrazolium (NBT) staining intensities as well as malondialdehyde (MDA) content were significantly higher in *osmads31* mutants and significantly lower in *OE* lines. Transcriptome analysis of WT and *osmads31* mutants under salt stress conditions, followed by Gene Ontology (GO) enrichment of the identified differentially expressed genes (DEGs), revealed the enrichment of genes encoding protein kinases, CATs, and transcription factors. Kyoto Encyclopedia of Genes and Genomes (KEGG) enrichment analysis identified several key pathways including carbon metabolism, amino acid biosynthesis, metabolic pathways, glycolysis/gluconeogenesis, lipid metabolism, and plant hormone signal transduction. Furthermore, weighted gene co-expression network analysis (WGCNA) of the DEGs demonstrated that *OsMADS31* enhances salt tolerance by upregulating antioxidant-related genes, activating antioxidant enzymes, and reducing oxidative damage. Our results conclusively show that *OsMADS31* improves salt tolerance in rice.

## 1 Introduction

Rice (*Oryza sativa* L.) is a globally vital staple crop, providing food for over half of the world’s population and playing a critical role in global food security systems (Sasaki and Burr, 2000; Wing et al., 2018). With the current global population exceeding 8 billion and projected to reach 9.7 billion by 2050, food demand will necessitate a 60% increase in crop production (Bailey-Serres et al., 2019; Hickey et al., 2019). Excessive soil salinity, an environmental stressor affecting approximately 7% of the land area globally, threatens the sustainability of crop production worldwide (Yang and Guo, 2018a). Moreover, rapid urbanization and industrialization have led to annual reductions in the size of arable land. Thus, the growing disparity between population growth and food demand, exacerbated by shrinking farmland, makes food security a critical challenge in China (Qin and Huang, 2020). China possesses abundant inland saline-alkali lands and coastal tidal flats (Esteban et al., 2016; Naveed et al., 2018). These underutilized salt-affected areas hold significant potential for future development into arable land. Among cereal crops, rice is particularly sensitive to salt stress (Hussain et al., 2018; Ma et al., 2016). Therefore, increasing the salt tolerance of crops is an urgent priority in agricultural research.

Salt stress exerts profound adverse effects on plants, disrupting physiological and biochemical processes throughout their entire life cycle from germination to senescence (Lodeyro and Carrillo, 2015; Muranaka et al., 2002; Murphy et al., 2003). Salt stress primarily induces osmotic stress and ionic stress, both of which can trigger oxidative stress in plant cells (Lodeyro and Carrillo, 2015; Zhu, 2016). To mitigate salt-induced damage, plants activate regulatory mechanisms to sustain cell growth and expansion (Fricke et al., 2004; Munns et al., 2000; Sackey et al., 2025). Salt stress rapidly induces osmotic stress in plants, since salt accumulation disrupts water and solute supply (Fricke et al., 2004; Munns et al., 2000; Qin and Huang, 2020; Zhao et al., 2020). Osmotic stress, in turn, triggers rapid stomatal closure in leaves, reducing CO_2_ uptake and inhibiting photosynthesis (Fricke et al., 2004). Excessive accumulation of sodium (Na^+^) and chloride (Cl^-^) ions in plant cells leads to premature leaf senescence and, in severe cases, plant death (Wegner et al., 2011; Yang and Guo, 2018a; Yang and Guo, 2018b). Elevated Na^+^ levels inhibit enzyme activity, disrupting metabolic processes such as the carbon cycle required for photosynthesis and other physiological pathways (Munns and Tester, 2008; Wu et al., 2018). When cytosolic Na^+^ concentrations exceed a critical threshold, the uptake and transport of essential elements, including potassium (K^+^), calcium (Ca²^+^), and zinc (Zn²^+^) is disrupted (Cheeseman, 2013; Iqbal et al., 2018; Munns et al., 2016; Razzaq et al., 2020; Seifikalhor et al., 2019; Shabala and Pottosin, 2014). Beyond osmotic and ionic stress, salt stress also induces the accumulation of reactive oxygen species (ROS) in plant cells, damaging cellular structures and macromolecules such as DNA, lipids, and enzymes (Ahanger et al., 2017; Miller et al., 2010). Under salt stress, plants must regulate ion homeostasis, osmotic balance, and oxidative stress responses to alleviate damage (Yang and Guo, 2018a; Yang and Guo, 2018b).

MADS-box genes encode transcription factors that regulate nearly all major aspects of land plant life. The MADS-box gene family participates in diverse biological processes, including vegetative development, flowering, and seed/fruit development (Saha et al., 2015), and has been extensively studied owing to its critical role in eukaryotic transcriptional regulation (de Folter and Angenent, 2006; Gramzow et al., 2010; Kaufmann et al., 2005). A defining feature of MADS-box genes is their highly conserved ~180-bp DNA sequence, which encodes the DNA-binding MADS domain (Kaufmann et al., 2005). MADS-box genes are classified as Type I or Type II, with Type II genes (termed MIKC-type MADS-box genes) predominantly governing floral organ identity (Kwantes et al., 2012; Smaczniak et al., 2012; Theissen et al., 2016). MIKC-type MADS-box transcription factors can form heterotetramers by assembling with closely related proteins. The resulting protein complexes bind to two CArG-box motifs within the same DNA loop, enhancing precise target gene recognition, and orchestrate key developmental processes in flowering plants, with distinct roles in vegetative growth and fruit development (Kappel et al., 2023). Notably, MIKC-type proteins generate intricate intra-family interaction networks, leveraging their unique ability to form multiprotein complexes comprising two or more homologous proteins that collectively function as transcriptional regulators.

MADS-box genes, as crucial regulatory factors, play pivotal roles in modulating plant developmental processes (Thompson et al., 2009; Yu et al., 2014b) and mediating hormone regulation and abiotic stress responses. In rice, genes belonging to the AGL17-like clade, including *OsMADS25*, *OsMADS27*, and *OsMADS57*, are nitrate-inducible (Puig et al., 2013; Yu et al., 2014a). Among these genes, *OsMADS25* and *OsMADS27* are critical for salt stress responses (Chen et al., 2018a; Xu et al., 2018); while *OsMADS25* regulates root growth and confers salt tolerance via abscisic acid (ABA) signaling and ROS scavenging (Wu et al., 2019), *OsMADS27* governs root development and osmotic stress adaptation, with its overexpression enhancing salt tolerance (Chen et al., 2018a). MADS-box transcription factor 27 (*mads27*) mutant, nitrate reductase-dependent nitric oxide production mediates nitrate-conferred salt tolerance in rice seedlings (Teng et al., 2025). Overexpression of the AGL17-like gene *OsMADS57* has been shown to improve seed germination and root growth in both Arabidopsis and rice under salt stress, significantly boosting salinity resistance through antioxidant system activation (Wu et al., 2021). OsMADS57 also enhances cold tolerance by binding to the *OsWRKY94* promoter (Chen et al., 2018b; Wu et al., 2019). In rice, *OsMADS26* acts as an upstream negative regulator of stress-related genes, suppressing pathogen resistance and drought tolerance (Khong et al., 2015), whereas *OsMADS87*, a heat-sensitive imprinted gene associated with syncytial endosperm, participates in thermosensitivity (Chen et al., 2016). Stress sensing and signaling pathways are essential for plant survival under adverse conditions. Investigating the adaptive strategies and gene networks of plant species that lead to high-salinity tolerance may facilitate the development and transfer of salt-resistant traits to major crops (Isayenkov, 2019). However, mechanistic insights into the roles of most MADS-box genes in salt stress responses in rice remain limited.

Soil salinity, a prevalent abiotic stress factor, adversely affects rice growth and yield (Wang et al., 2018). Investigating MADS-box genes involved in abiotic stress responses in rice holds significant value, as it could help to facilitate the enhancement of stress tolerance through genetic engineering. In our previous work, we identified two salt stress-responsive jacalin-related lectin (JRL) rice genes, *OsJRL45* and *OsJRL40* (Gao et al., 2023a). Subsequent physiological and biochemical analyses confirmed the role of *OsJRL45* and *OsJRL40* in salt stress tolerance (Gao et al., 2023b, Gao et al., 2023c; Yin et al., 2024). Using a yeast two-hybrid screen, we further isolated the MADS-box transcription factor OsMADS31, which interacts with OsJRL40 and is hypothesized to participate in the salt stress response. While the MADS-box transcription factor family plays crucial roles in plant growth and development, the function of *OsMADS31* in abiotic stress responses remains unclear. This study aimed to investigate whether *OsMADS31* responds to salt stress. By generating *OsMADS31* knockout and overexpression plants and conducting gene expression analysis, we found that *OsMADS31* expression was strongly induced by NaCl, indicating that it has a critical role under salt stress conditions.

## 2 Materials and methods

### 2.1 Plant materials and growth conditions

WT rice (*Oryza sativa* L) cultivar Nipponbare (Nip), along with *OsMADS31* knockout (*osmads31*) and overexpression (*OE*) lines generated in the Nip background, were used in this study. Seeds were germinated and cultivated hydroponically following established protocols. Plants were grown in a greenhouse under controlled conditions: 30°C day/28°C night temperature, 70% relative humidity, 14-h light/10-h dark photoperiod, and 600 μmol m^-^² s^-^¹ light intensity.

### 2.2 Bioinformatics analysis

#### 2.2.1 Sequence alignment and visualization

Nucleotide sequences of the MIKC-type MADS-box genes of rice (*Oryza sativa* L) were retrieved from the Rice Genome Annotation Project (https://rice.uga.edu/). Subsequently, the deduced amino acid sequences of MIKC-type OsMADS-box transcription factors were aligned using MEGA 7.0.26 with the ClustalW algorithm. The aligned sequences were exported in FASTA format and visualized using ESPript 3.0 (https://espript.ibcp.fr/ESPript/ESPript/), with customized parameters for color schemes, font size (10 pt), residues per line (60), canvas dimensions, and output format (PNG). Sequence logos of conserved domains were generated using WebLogo 3 (https://weblogo.threeplusone.com/).

#### 2.2.2 Phylogenetic analysis

Amino acid sequences of MIKC-type MADS-box proteins of rice, *Arabidopsis thaliana*, maize (*Zea mays*), and wheat (*Triticum aestivum*) were downloaded from the National Center for Biotechnology Information (NCBI, https://www.ncbi.nlm.nih.gov/). After ClustalW2-based multiple sequence alignment, a maximum-likelihood (ML) phylogenetic tree with 1,000 bootstrap replicates was constructed using MEGA 7.0.26 (Kumar et al., 2018) and annotated using Evolview (https://www.evolgenius.info/).

#### 2.2.3 Conserved domain analysis

Conserved motifs in the OsMADS31 amino acid sequence were predicted using MEME5.1.7 (https://meme-suite.org/meme/), with 12 motifs spanning the full-length protein. Domain architecture was analyzed using the NCBI Conserved Domains Database (CDD; https://www.ncbi.nlm.nih.gov/Structure/bwrpsb/bwrpsb.cgi), and results were integrated with the phylogenetic tree using TBtools v2.142 for comparative visualization of evolutionary relationships and structural features (Chen et al., 2023).

### 2.3 Generation of mutant and transgenic lines

The CRISPR/Cas9 system was employed to generate *osmads31* knockout mutants. Two 20-bp target sequences (5’-GTGATTGTGTTCTCAGGCAC-3’ and 5’-GCACCCGTTTTGAGGAGATG-3’) were selected from the *OsMAS31* coding sequence using established protocols (Miao et al., 2013). The *osmads31* knockout plasmids were introduced into the Nip cultivar via *Agrobacterium*-mediated transformation. Transgenic plants were selected for hygromycin resistance and genotyped by PCR using knockout identification primers (*osmads31*-F/R, **Supplementary Table 1**). Two independent *osmads31* knockout mutant lines (*KO1* and *KO2*) were obtained, and potential off-target effects were evaluated using CRISPR-P (http://cbi.hzau.edu.cn/cgi-bin/CRISPR) (Liu et al., 2017).

To generate *OsMADS31* overexpression (*OE*) lines, the *OsMADS31* open reading frame (ORF) was amplified from Nip cDNA using overexpression identification primers (*OsMADS31OE*-F/R, **Supplementary Table 1**) and then cloned into the pCAMBIA1390-Ubi vector. To generate *pOsMADS31::GUS* transgenic plants, a 2-kb fragment of the *OsMADS31* promoter (upstream of ATG) was amplified from the Nip genomic DNA using GUS identification primers (*OsMADS31*GUS-F/R, **Supplementary Table 1**) and cloned into pCAMBIA1301-Ubi. All constructs were verified by sequencing (Tsingke Biotechnology) and subsequently introduced into *Agrobacterium tumefaciens* strain EHA105 by electroporation. The transformed *Agrobacterium* cells were then used to transfect Nip plants as described previously (Hiei et al., 1994).

### 2.4 RNA extraction and gene expression analysis

Total RNA was extracted from rice cultivar Nip plants using the TRIzol Reagent (Invitrogen, Waltham, MA, USA). To carry out the spatial expression analysis of *OsMADS31*, total RNA was isolated from root and shoot in Nip three-leaf stage seedlings, and different tissues of Nip heading stage including root, node, leaf, culm, anther, and young spike. The isolated total RNA was reverse-transcribed into cDNA. Subsequently, RT-qPCR was performed on the ABI PRISM 7500 system (Applied Biosystems) using Takara RR420A reagents and gene-specific primers designed with Oligo 7 (Sangon Biotech, Shanghai). *OsActin* served as the internal control gene.

Seedlings of the WT as well as *osmads31* mutant and *OsMADS31* overexpression lines were treated with 6‰ NaCl at the three-leaf-stage. RNA was extracted from the salt-treated seedlings using TRIzol Reagent, and cDNA was synthesized using HiScript II QRT SuperMix (+gDNA wiper) (Vazyme R223-01). Then, qPCR was conducted using ChamQ Universal SYBR qPCR Master Mix (Vazyme Q711-02), with *OsActin* serving as the reference gene. Relative gene expression levels were calculated using the 2^−ΔΔCt^ method (Livak and Schmittgen, 2001) and presented as mean ± SD. All primers used in this study are listed in **Supplementary Table 2**.

### 2.5 GUS staining assay

GUS staining was performed as previously described (Li et al., 2015). Briefly, tissues of *pOsMADS31::GUS* transgenic rice plants were incubated in GUS staining solution overnight at 37°C. Plant tissues were subsequently destained using 75% ethanol to remove chlorophyll. Then, the tissues were examined and photographed using the Olympus SZX7 microscope.

### 2.6 ROS determination

The accumulation of O_2_^•-^ and H_2_O_2_ in rice plants was determined by staining with nitroblue tetrazolium chloride (NBT) and 3,3’-diaminobenzidine (DAB), respectively, as described previously (Huang et al., 2019; Jambunathan 2010). Briefly, the second leaves were collected from the *KO*, *OE* and WT plants before and after the 6‰ NaCl treatment. To perform NBT staining, 3-cm segments of leaves were prepared, vacuum-infiltrated with the NBT solution (0.1%, pH 7.8) for 30 min, and incubated at room temperature for 24 h. To perform DAB staining, leaf samples were vacuum-infiltrated with the DAB solution (1 mg/mL in 10 mM Na_2_HPO_4_ containing 0.05% Tween-20, pH 3.8) for 30 min and then incubated at 37°C with shaking for 48 h. Both the NBT- and DAB-stained samples were destained with 95% ethanol and photographed under a stereomicroscope (Nikon XAZ100).

### 2.7 Subcellular localization analysis

To determine the subcellular localization of OsMADS31, the coding sequence (CDS) of *OsMADS31* (excluding the stop codon) was amplified from the Nip genomic DNA using gene-specific primers (**Supplementary Table 1**). The PCR product was cloned into the pCAMBIA1390-GFP vector. The resulting *35S::OsMADS31-GFP* construct was transiently expressed in rice protoplasts via polyethylene glycol (PEG)-mediated transformation (Wang et al., 2021). GFP signal was visualized using the Zeiss LSM880 confocal microscope (Wang et al., 2022a).

### 2.8 Salt stress treatment of transgenic plants

After treatment with different concentrations of NaCl, the germination rate of seeds was counted on the seventh day. Transgenic and WT seeds were incubated on Murashige and Skoog (MS) medium containing varying NaCl concentrations for 12 days, and root and shoot lengths were measured subsequently.

Uniform, plump rice seeds were surface-sterilized with 0.3% sodium hypochlorite for 20 min, rinsed three times with distilled water, and placed on moist filter paper in Petri dishes at 37°C. The germinated seeds were transferred to 96-well trays containing culture solution, replaced every 4 days. After growing to the three-leaf stage (~2 weeks) under standard hydroponic conditions, seedlings were exposed to 6‰ NaCl solution for 6 days. Subsequently, the seedlings were allowed to recover for 6 days in normal solution before calculating the survival rate.

### 2.9 Measurement of physiological parameters

SOD, POD, and CAT activities as well as MDA and Pro contents were measured using the respective kits, as described previously (Gao et al., 2023a, Gao et al., 2023b, Gao et al., 2023c). Measurements were taken both pre-treatment and at 24 h post-salt-stress treatment. Three biological replicates were performed, and data were expressed as mean ± SD.

### 2.10 RNA sequencing analysis

WT and *osmads31* mutant transgenic seedlings at the three-leaf stage were sampled pre-treatment and at 24 h after 6‰ NaCl exposure. Three biological replicates were performed per genotype, and the seedlings were flash-frozen in liquid nitrogen and submitted to Genedenovo Biotechnology Co., Ltd (Guangzhou, China) for RNA-seq. The obtained transcriptomic data were analyzed using established methods (Gao et al., 2023b, Gao et al., 2023c; Zhang et al., 2020). Additional data analysis was conducted using the Genedenovo Biotechnology Platform Omicsmart (https://www.omicsmart.com). The cDNA libraries were sequenced on the Illumina sequencing platform by Genedenovo Biotechnology Co., Ltd (Guangzhou, China).

### 2.11 Interaction network analysis

Relative expression levels of differentially expressed genes (DEGs), identified through RNA-seq, were subjected to log-transformation and normalization. Subsequently, the gene expression data were examined using correlation analysis, weighted gene co-expression network analysis (WGCNA)-based network construction, and Cytoscape visualization (Hu et al., 2016). Protein-protein interactions were predicted using STRING (https://cn.string-db.org/).

### 2.12 Statistical analysis

Data are presented as the mean ± SD of three or more independent replicates. The mean of replicates for each experiment was calculated using the PASW Statistics 18 software. Statistical significance was determined using one-way ANOVA and Student’s *t*-test at *p* < 0.05. Bar charts were created using GraphPad Prism 10.3.1. Heatmaps were constructed and drawn using TBtools-II v.2.142 (Chen et al., 2023).

## 3 Results

### 3.1 Conservation and amino acid sequence analysis of MIKC-type MADS domains in rice

To characterize the conserved features of MIKC-type MADS domains in rice, we performed multiple sequence alignment of their amino acid sequences. The results showed that MADS domains of the DNA binding domain are highly conserved among the 61 MIKC-type MADS-box transcription factors found in rice (**Supplementary Figure 1**). According to previous studies, MIKC-type MADS domains exist in two lineages (Type I and Type II) of MADS-box genes across plants, animals, and fungi. Notably, the majority of plant MADS-box genes belong to the Type II lineage and contain three additional domains compared with Type I genes: an intervening (I) domain, a keratin-like coiled-coil (K) domain, and a variable-length C-terminal (C) domain; these three domains together form the plant-specific MIKC-type structure (Nam et al., 2003). The conserved nature and distinctive characteristics of MIKC-type MADS domains provide important clues for determining the functional roles of MADS-box transcription factors in plant development and stress responses.

### 3.2 Analysis of conserved sequences and domains of the *OsMADS31* gene in rice

The *OsMADS31* CDS comprises 723 base-pairs and is predicted to encode a 240-amino acid protein. The amino acid sequence of OsMADS31 contains both the MADS and K-box domains. The MADS domain is involved in DNA binding and protein dimerization. Notably, MADS-box family proteins typically function as dimers, with their primary DNA-binding element consisting of an antiparallel coiled-coil formed by two amphipathic α-helices from each subunit (Norman et al., 1988). The MADS domain is generally associated with the K-box region. Most plant MADS-box proteins possess the typical MIKC structure, which mediates dimerization and cofactor binding, serving as the primary determinant for DNA binding. The K-box protein interaction domain, which mediates heterodimerization of MIKC-type MADS-box proteins, contains several heptad repeats with hydrophobic amino acids at the first and fourth positions. This suggests that the K-box domain forms three amphipathic α-helices, designated as K1, K2, and K3 (Yang et al., 2003). Analysis of conserved motifs among the 61 highly conserved MIKC-type MADS-box proteins identified in rice (**Supplementary Figure 2**) revealed that all of these 61 MADS-box proteins contain both the MADS and K-box domains, with sequence logos showing 12 conserved amino acid residue motifs. Phylogenetic analysis demonstrated that the rice gene *OsMADS31* is evolutionarily closely related to its orthologs in maize (**Supplementary Figure 3**).

### 3.3 Examination of *OsMADS31* gene expression and OsMADS31 protein localization

**Figure 1:**
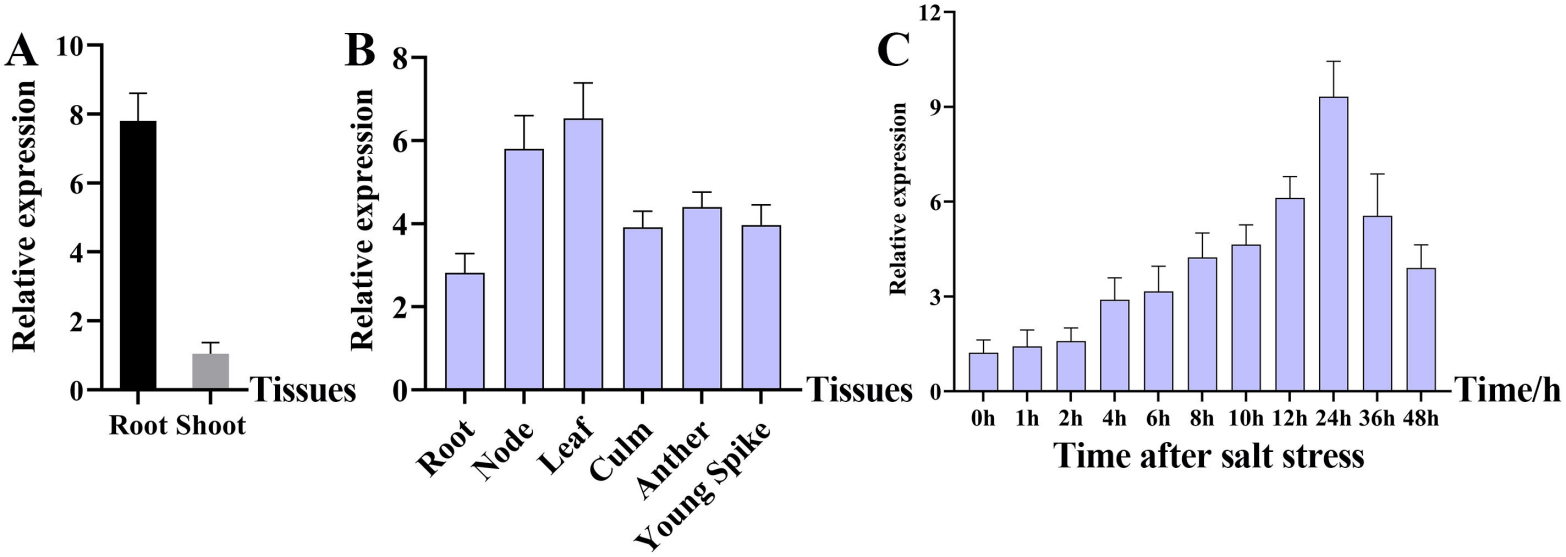
Characterization of the *OsMADS31* gene using RT-qPCR. **(A)** Expression analysis of *OsMADS31* at root and shoot in rice three-leaf stage seedlings by RT-qPCR, **(B)** expression analysis of *OsMADS31* at different parts in rice heading stage by RT-qPCR, **(C)** expression analysis of *OsMADS31* at different times under salt stress in rice three-leaf stage seedlings by RT-qPCR.

The expression of *OsMADS31* was examined in the root and shoot of WT Nip three-leaf stage seedlings (**Figure 1A**), and different tissues (roots, nodes, leaves, stems, anthers, and young panicles) of WT Nip heading stage (**Figure 1B**). The results of RT-qPCR analysis showed that the expression of *OsMADS31* began to increase at 4 h after the salt stress treatment at the three-leaf-stage of WT Nip and peaked at 24 h, with expression levels reaching approximately 10-fold higher than the baseline level (**Figure 1C**). To further examine *OsMADS31* expression, a vector containing the *OsMADS31* promoter-driven *GUS* reporter gene was constructed and transformed into Nip. GUS staining of the resulting transgenic plants demonstrated GUS activity in the coleoptile of germinating seeds as well as in whole seedlings, stems, leaves, anthers, and young panicles (**Supplementary Figure 4**). Quantitative analysis indicated higher expression in leaves and stem nodes, and the cross-sections of stained tissues revealed deeper staining in vascular bundles. These results suggest that *OsMADS31* is expressed in multiple tissues and at different developmental stages, implying its functional importance in rice growth.

**Figure 2:**
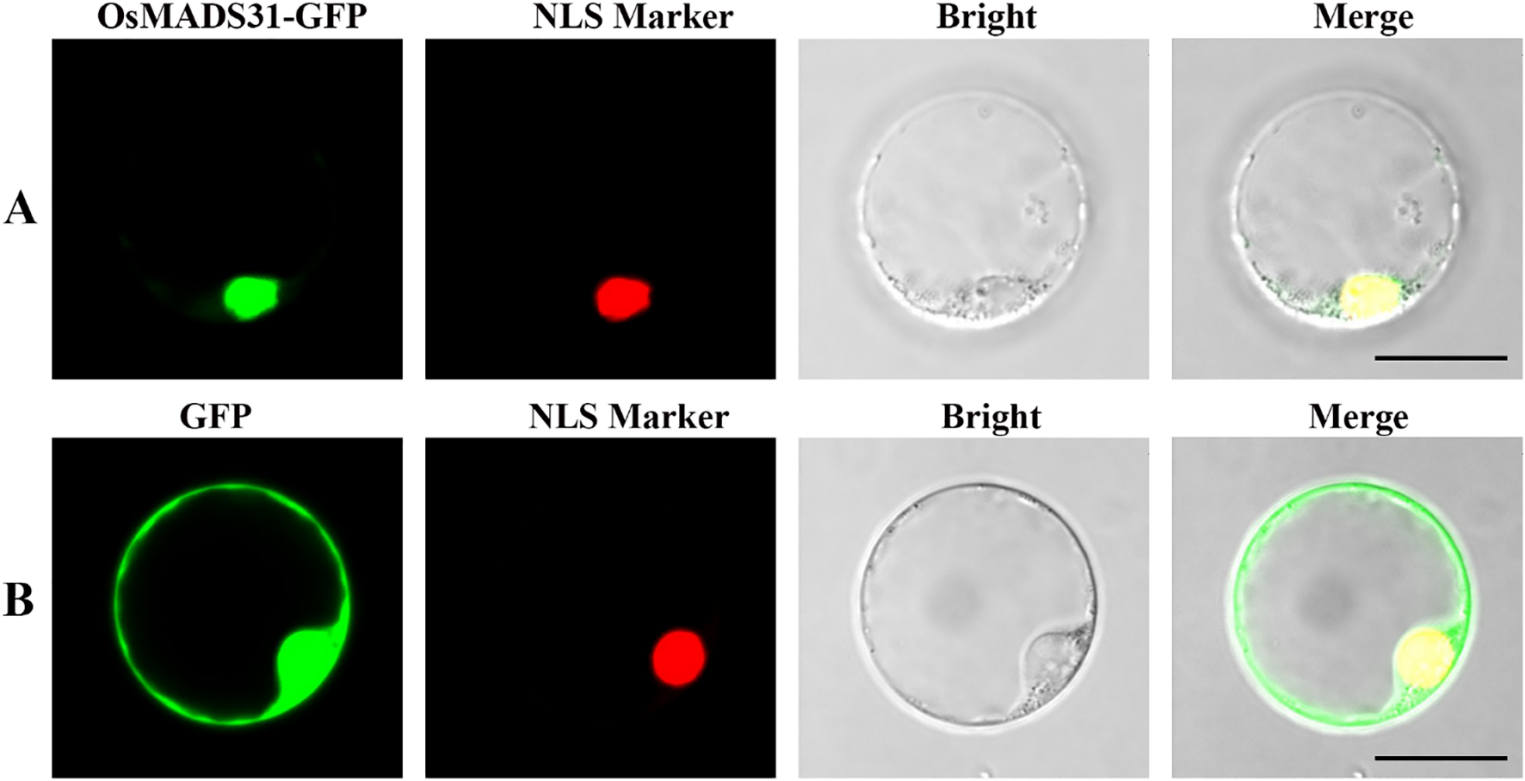
Subcellular localization analysis of OsMADS31 in transgenic rice protoplasts using confocal fluorescence microscopy. **(A, B)** Green fluorescence signal and nuclear localization signal (NLS) in rice protoplasts carrying the *35S::OsMADS31-GFP* construct **(A)** and *35S::GFP* construct **(B)**. Bright, brightfield, Merge, fluorescence channel and brightfield overlay. Scale bars = 10 μm.

We also examined the subcellular localization pattern of OsMADS31. Online prediction tools indicated that OsMADS31 is nuclear-localized. These results were experimentally verified (**Figure 2**).

### 3.4 Analysis of *osmads31* knockout mutant and *OsMADS31* overexpression lines at maturity

To further investigate whether *OsMADS31* is involved in salt stress tolerance, a vector for knocking-out *OsMADS31* expression was constructed using the CRISPR/Cas9 technology and introduced into Nip via *Agrobacterium*-mediated transformation. The resultant *osmads31* knockout mutants were verified by sequencing and off-target cleavage detection (**Supplementary Figure 5A**). Concurrently, *OsMADS31* overexpression lines were generated in the Nip background. Finally, two knockout mutants (*KO1* and *KO2*) and two overexpression lines (*OE1* and *OE2*) were selected, based on the expression levels of *OsMADS31* determined by RT-qPCR (**Supplementary Figure 5B**). All four genotypes were propagated to the T_2_ generation, and homozygous knockout mutant and transgenic plants were used for subsequent experiments.

Next, we investigated the effects of the knockout mutation of *OsMADS31* on the phenotype and agronomic traits of homozygous T_2_-generation *KO* and *OE* plants at maturity, with the WT plants serving as controls. Knockout mutant plants exhibited significant reductions in grain length, grain width, plant height, seed-setting rate, 1000-grain weight, panicle length, and grains per panicle compared with WT plants. Conversely, overexpression lines displayed significant increases in panicle length and grain number per panicle compared with the WT (**Supplementary Figure 6** & **7**). These results demonstrate that the knockout mutation of *OsMADS31* affects rice seed development.

### 3.5 Evaluation of the salt stress tolerance of *osmads31* knockout mutant and *OsMADS31* overexpression lines at the seed germination stage

**Figure 3:**
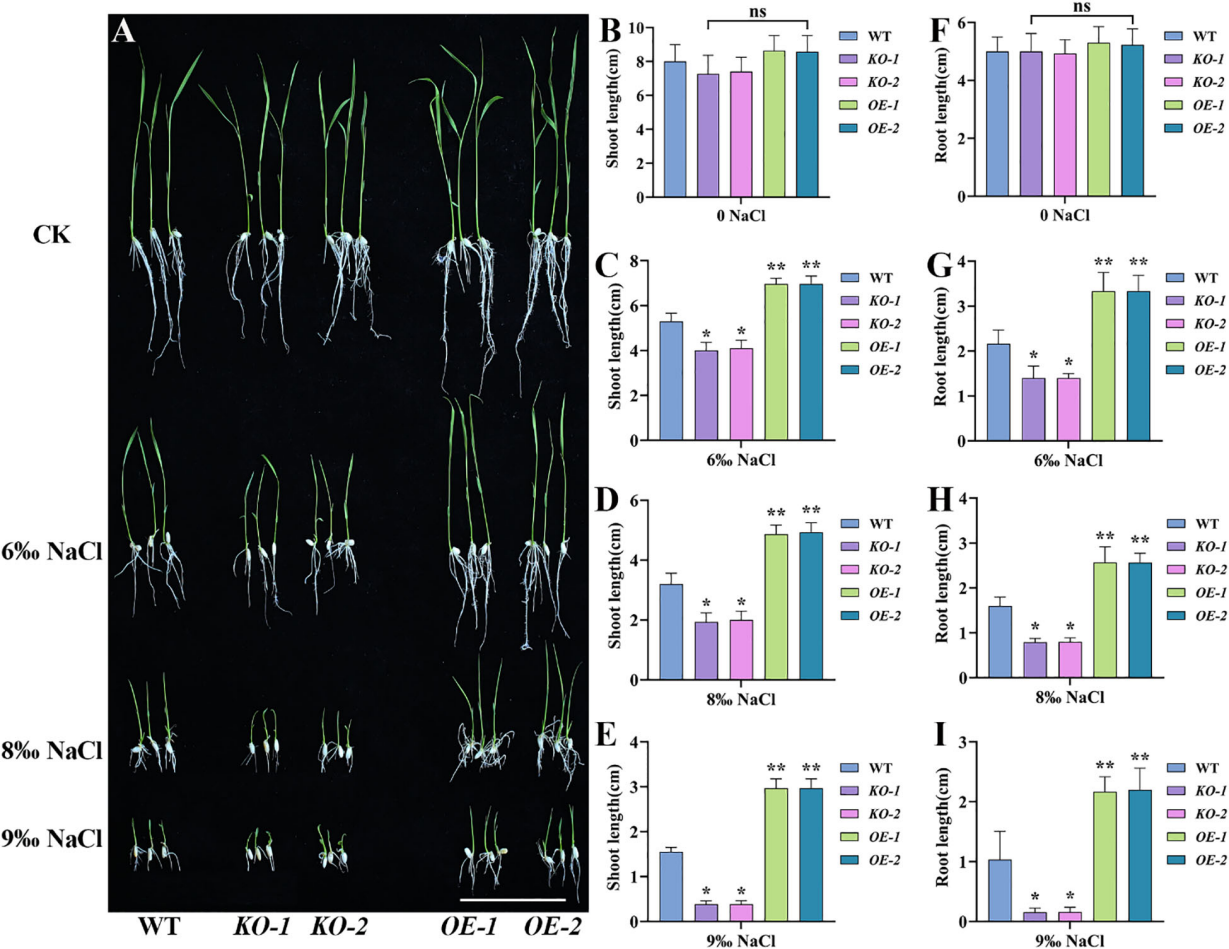
Analysis of seed germination and seedling growth in *osmads31* knockout mutants (*KO-1* and *KO-2*), *OsMADS31* overexpression lines (*OE-1* and *OE-2*), and the WT under salt stress. **(A)** Phenotypic analysis of seed germination across the three genotypes under different NaCl concentrations. Scale bar = 5 cm. **(B-I)** Quantitative analysis of shoot length **(B-E)** and root length **(F-I)** of WT, *KO*, and *OE* plants under different NaCl concentrations. Data represent the mean ± SD of three independent replicates. Asterisks indicate statistically significant differences compared with the WT (*P < 0.05, **P < 0.01). ns, no significant difference.

We further investigated the salt stress tolerance of *osmads31* knockout mutant and *OsMADS31* overexpression plants by performing seed germination assays. Uniformly plump seeds of the WT, *osmads31* knockout mutants (*KO1* and *KO2*), and *OsMADS31* overexpression lines (*OE1* and *OE2*) were germinated on MS medium containing no NaCl (control; CK) or different NaCl concentrations (6‰, 8‰, 9‰) (**Figure 3A**). Under normal conditions (CK), seeds of all genotypes showed normal germination and growth, with no significant differences. At 6‰ and 8‰ NaCl concentrations, compared with the WT, *KO1* and *KO2* seedlings exhibited significantly reduced shoot and root lengths, whereas *OE1* and *OE2* seedlings showed significantly increased shoot and root lengths (**Figure 3B-I**). At 9‰ NaCl concentration, although *KO1* and *KO2* seeds could germinate, their seedlings displayed nearly no root or shoot growth; by contrast, *OE1* and *OE2* seedlings were maintained growth of both shoots and roots (**Figure 3**). These results demonstrate that *OsMADS31* plays a crucial role in salt stress tolerance during seed germination.

### 3.6 Analysis of the salt stress tolerance of rice seedlings at the three-leaf stage

**Figure 4:**
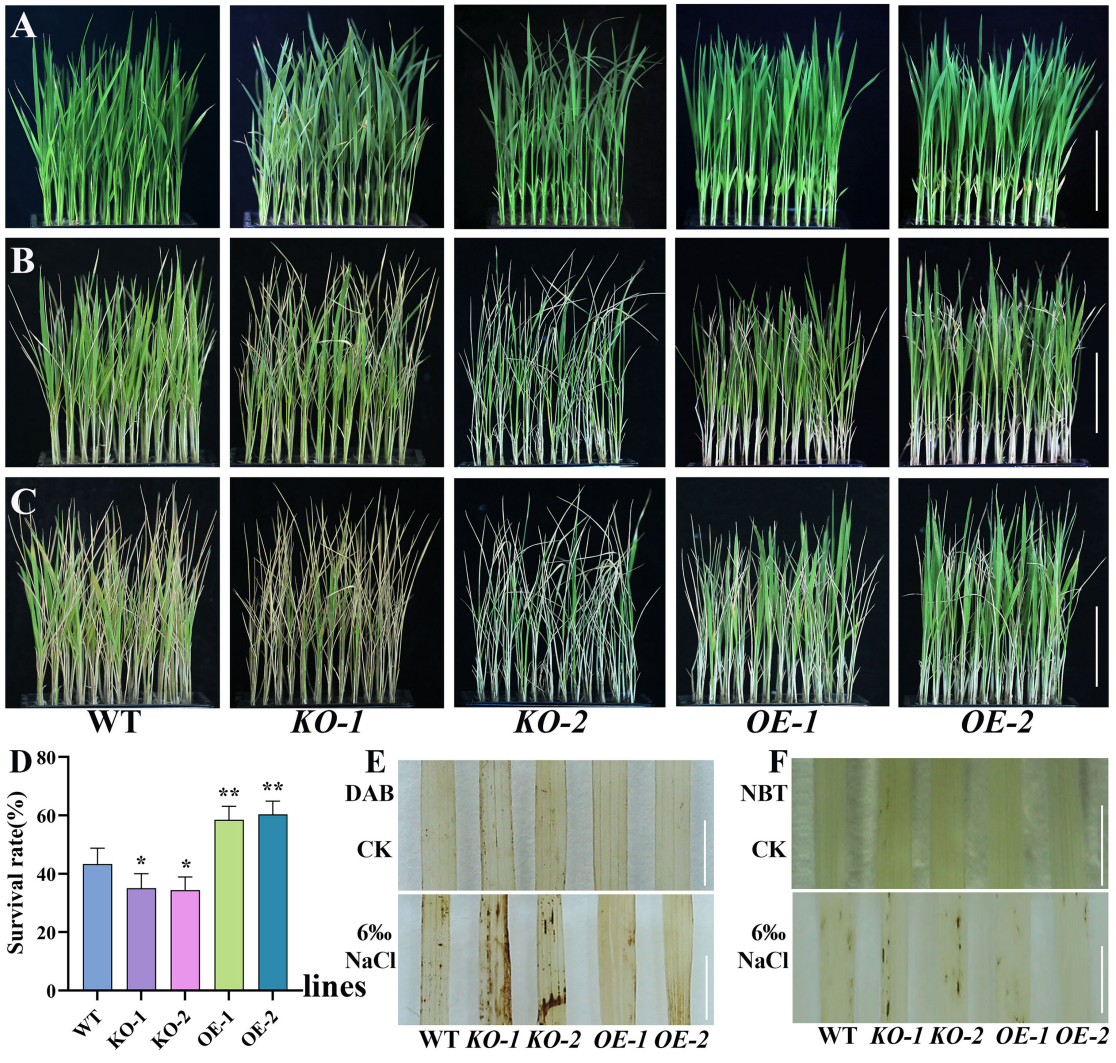
Analysis of the salt stress tolerance of the WT, *osmads31* knockout mutants (*KO-1* and *KO-2*), and *OsMADS31* overexpression lines (*OE-1* and *OE-2*) at the three-leaf stage. **(A-C)** Phenotypic analysis of WT, *KO*, and *OE* plants before salt stress **(A)**, at 6 days after treatment with 6‰ NaCl **(B)**, and at 6 days post recovery **(C)**. **(D)** Survival rates of plants after recovery from salt stress treatment. **(E)** DAB and **(F)**NBT staining of leaves. Asterisks indicate statistically significant differences compared with the WT (*P < 0.05, **P < 0.01). Scale bars = 5 cm.

To further evaluate the salt stress tolerance of the WT, *osmads31* knockout mutants, and *OsMADS31* overexpression lines, seedlings at the three-leaf stage were subjected to 6‰ NaCl treatment for 6 days, followed by a 6-day recovery period, and then analyzed for phenotypic differences (**Figure 4A-C**). The *KO* mutant seedlings exhibited wilting and growth retardation under salt stress, with significantly lower survival rates than the WT after recovery; by contrast, *OE* plants outperformed the WT in growth and showed significantly higher survival rates (**Figure 4D**). These results demonstrate that *OsMADS31* enhances salt stress tolerance in rice.

### 3.7 *OsMADS31* enhances the antioxidant capacity of rice under salt stress

**Figure 5:**
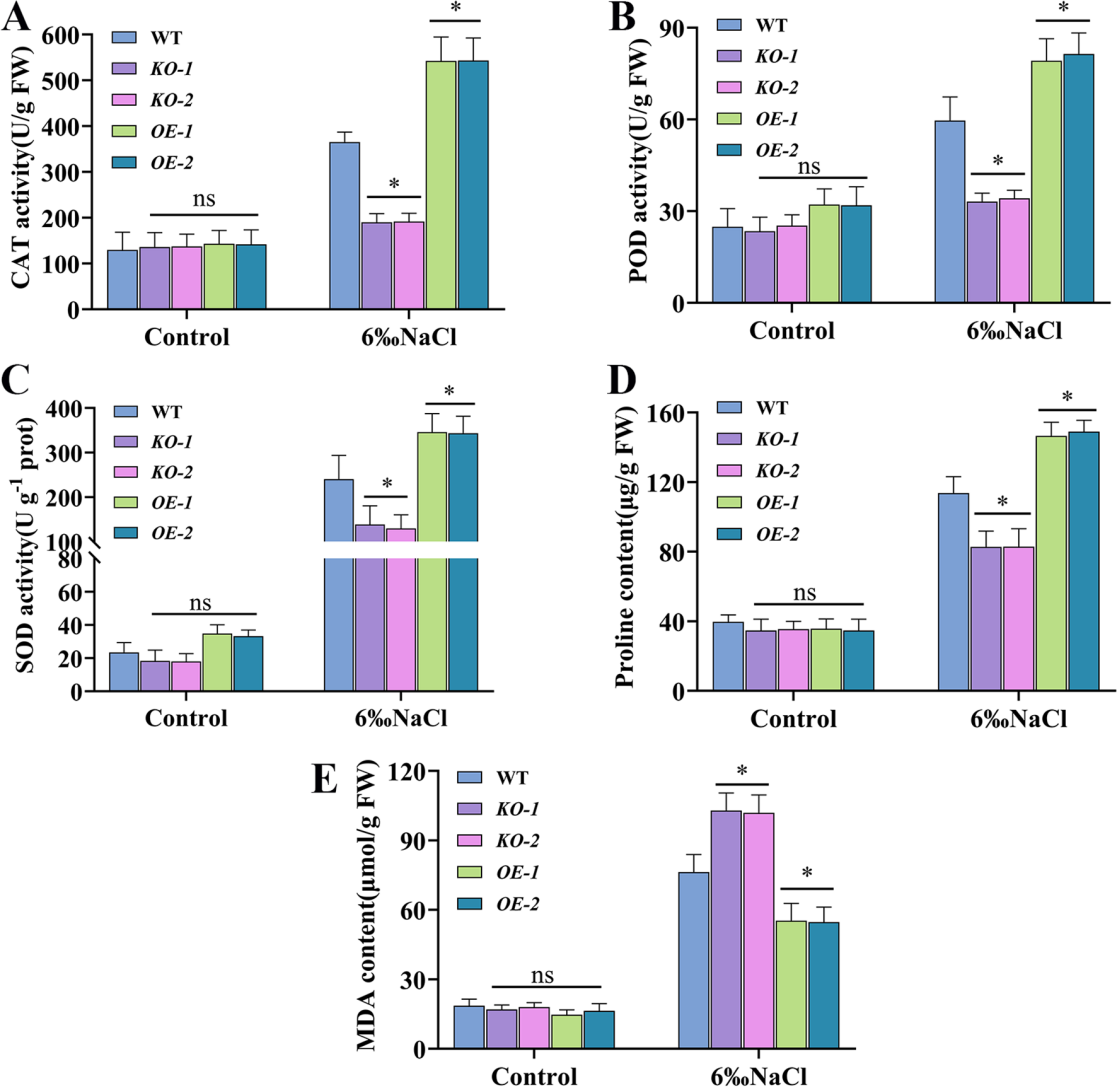
Analysis of the physiological parameters of *osmads31* knockout mutant (*KO-1* and *KO-2*), *OsMADS31* overexpression (*OE-1* and *OE-2*), and WT plants at the three-leaf stage before and after treatment with 6‰ NaCl for 24 (h) **(A-C)** Activities of CAT **(A)**, POD **(B)** and SOD enzymes **(C, D)** Proline quantification. **(E)** MDA quantification. Data represent the mean ± SD of three independent replicates. Asterisks indicate statistically significant differences compared with the WT (*P < 0.05). ns, no significant difference. CAT, catalase; POD, peroxidase; SOD, superoxide dismutase; Pro, proline; MAD, malondialdehyde; DAB, 3,3’-diaminobenzidine; NBT, nitroblue tetrazolium chloride.

Next, to determine the defense response of *osmads31* knockout mutants and *OsMADS31* overexpression lines to salt stress, we measured the activities of antioxidant enzymes (CAT, POD, and SOD) as well as the contents of MDA and Pro in WT, *KO*, and *OE* plants at the three-leaf stage before and after treatment with 6‰ NaCl for 24 h. Compared with the WT, *KO* mutants exhibited significantly lower CAT, POD, SOD activities and Pro content (**Figures 5A-D**), but significantly higher MDA content (**Figure 5E**); by contrast, *OE* mutants showed the opposite trends. We also determined the accumulation of ROS in all three genotypes before and after the salt stress treatment by performing DAB and NBT staining of leaves, followed by destaining. The intensity of NBT and DAB staining was significantly higher in *KO* mutants than the WT (**Figures 4E, F**). Together, these results indicate that knocking-out *OsMADS31* expression reduces antioxidant enzyme activities and disrupts the homeostasis of related physiological parameters under salt stress in rice.

### 3.8 Identification of genes and interaction networks regulated by *OsMADS31*

To further elucidate the role of *OsMADS31* in salt stress response at the molecular level, we performed Gene Ontology (GO) enrichment analysis of WT and *osmads31* knockout mutants before and after salt stress treatment DEGs. Comparative analysis of secondary bar plots revealed that, compared with the pre-treatment control (WT-vs-*KO* (CK)), salt-stressed samples (WT-vs-*KO* (Salt)) showed significantly fewer upregulated genes than downregulated genes. Notably, in the Biological Process category, genes involved in ‘response to stimulus’ were downregulated by more than twofold in WT-vs-*KO* (Salt) compared with WT-vs-*KO* (CK) (**Supplementary Figures 8A, C**). Volcano plot of differentially expressed genes after under salt stress (**Supplementary Figures 8E**). Comparative GO enrichment circle plots before and after the salt stress treatment demonstrated significant enrichment of genes in the Biological Process category (**Supplementary Figures 8B, D**). Bubble plot analysis further revealed that the WT-vs-*KO* (Salt) comparison was primarily enriched in GO terms such as ‘response to harmful substances’ and ‘metabolic processes’ (**Supplementary Figures 9A, C**). The directed acyclic graph (DAG) showed predominant enrichment of genes involved in ‘response to stimulus’ (**Supplementary Figures 9B, D**). Subsequent Gene Set Enrichment Analysis (GSEA) of ‘response to stimulus’-related genes after salt treatment highlighted processes related to salt stress response, while regulatory network analysis identified three key functional categories: protein kinases, catalases, and transcription factors (**Supplementary Figures 9E, F**). These results suggest that salt stress likely affects plant physiological responses by modulating specific gene expression patterns in rice.

Among the DEGs, 455 were upregulated and 756 were downregulated genes in *KO* mutants following salt treatment (**Supplementary Figure 10A, B**), suggesting that these DEGs may be directly associated with salt stress response. KEGG enrichment analysis identified the following top 10 pathways in WT-vs-*KO* (CK): ‘Biosynthesis of secondary metabolites, Cutin, suberin, and wax biosynthesis, Protein processing in the endoplasmic reticulum, Metabolic pathways, Glutathione metabolism, Carbon fixation in the calvin cycle, Photosynthesis-antenna proteins, Phenylpropanoid biosynthesis, Benzoxazinoid biosynthesis, and Betaine biosynthesis’ (**Supplementary Figure 10C**). Similarly, KEGG enrichment analysis of the WT-vs-*KO* (Salt) data showed enrichment of the following 10 pathways: ‘Phenylpropanoid biosynthesis, Biosynthesis of secondary metabolites, Plant hormone signal transduction, Pyruvate metabolism, Metabolic pathways, Alanine, aspartate and glutamate metabolism, Glycolysis/Gluconeogenesis, MAPK signaling pathway-plant, Plant-pathogen interaction, and Arginine and proline metabolism’ (**Supplementary Figure 10D**). KEGG hierarchy analysis of the top 20 pathways identified in WT-vs-*KO* (Salt) revealed carbon metabolism, amino acid biosynthesis and metabolic pathways as the three most affected pathways, with glycolysis/gluconeogenesis and lipid metabolism also potentially impacted. Subsequent GSEA revealed genes involved in plant hormone signal transduction (**Supplementary Figure 10E-G**). Among the genes downregulated under salt stress, several encoded transcription factors belonging to the NAC, MYB, ethylene-responsive factor (ERF), AP2/EREBP, basic helix-loop-helix (bHLH), and bZIP families (**Supplementary Figure 10H**). These results demonstrate that salt stress primarily affects the physiological and metabolic processes of plants by regulating genes involved in secondary metabolite biosynthesis, metabolic pathways, and plant hormone signal transduction.

To further investigate the relationship between *OsMADS31* and DEGs, we performed WGCNA. WGCNA is a systematic biological method used to analyze inter-gene relationships in expression data by constructing weighted networks based on co-expression patterns for identifying gene modules associated with specific phenotypes. In this study, WGCNA was conducted using 17,695 genes with relative expression levels > 1. The results revealed 20 distinct modules, with each module containing 3 to 7,836 genes. Among these, four modules (black, brown4, magenta, and pink) showed significant associations (**Supplementary Figure 11**). The identification of these modules provides crucial insights for understanding the mechanism of *OsMADS31*-mediated salt stress tolerance in rice.

**Figure 6:**
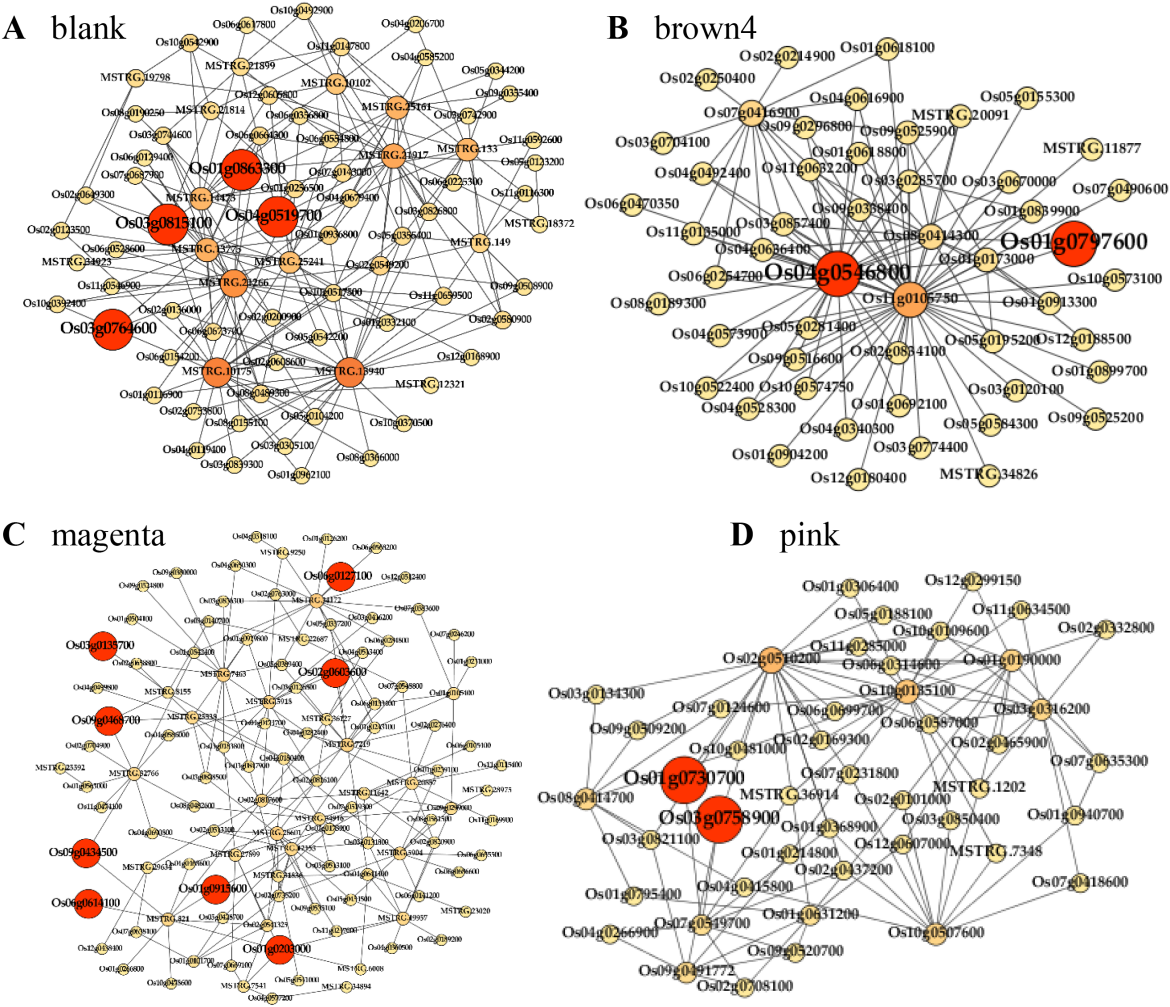
Analysis of co-expression interaction network genes in each module. **(A-D)** Gene co-expression networks for the black module (four genes) **(A)**, brown4 module (two genes) **(B)**, magenta module (eight genes) **(C)**, and pink module (two genes) **(D)**.

Gene regulatory network analysis enables the identification of hub genes within the regulatory network and facilitates functional prediction of unknown genes based on known gene functions. GO and KEGG enrichment analyses demonstrated that these modules were primarily involved in biosynthesis of secondary metabolites and metabolic pathways, consistent with previous KEGG enrichment findings. Although each module exhibited distinct KEGG pathways, a substantial number of genes participated in several common pathways including Biosynthesis of secondary metabolites, Plant hormone signal transduction, Metabolic pathways, and Peroxisome-related processes (**Supplementary Figure 12**). Co-expression network analysis was performed to identify hub (key) genes within each module and their interactions with genes in other modules (**Figure 6**). The black module contained 200 genes, including four hub genes (*Os03g0764600*, *Os03g0815100*, *Os04g0519700*, and *Os01g0863300*), encoding an HHO transcription factor, a stress-responsive NAC transcription factor, an auxin response factor, and a MYB family transcription factor, respectively. For example, the core gene *Os03g0815100* in the Black module encodes the stress-responsive NAC transcription factor (SNAC1). Studies have shown that overexpressing SNAC1 upregulates the expression of multiple stress-related genes, thereby enhancing salt tolerance in rice (Hu et al., 2006). The brown4 module comprised 100 genes, with three hub genes *(Os05g0195200*, *Os04g0546800*, and *Os01g0797600*) that encode a CCCH tandem zinc finger protein, an ethylene-responsive transcription factor, and an ERF, respectively. For example, the core gene *Os01g0797600* (ethylene-responsive transcription factor ERF3/OsAP37) in the Brown4 module, when its encoded protein OsAP37 is overexpressed in transgenic rice plants driven by the *OsCc1* promoter, exhibited enhanced salt tolerance during the vegetative growth stage (Oh et al., 2009). The magenta module included 200 genes, with eight hub genes (*Os01g0203000*, *Os06g0127100*, *Os01g0915600*, *Os03g0135700*, *Os09g0468700*, *Os02g0603600*, *Os09g0434500*, and *Os06g0614100*), encoding a BZR1 homolog, an AP2/EREBP transcription factor, a bHLH transcription factor, an ERF, a bZIP transcription factor, and two helix-loop-helix DNA-binding proteins. For example, the core gene *Os06g0127100* (AP2/EREBP transcription factor) in the Magenta module encodes dehydration-responsive element-binding proteins 1C, 1E, and 1G (DREB1C, DREB1E, DREB1G), which promote tolerance to salt stress in rice (Wang et al., 2022b). Another core gene in the Magenta module, *Os09g0434500* (ethylene-responsive factor OsBIERF), belongs to a family whose members OsBIERF1, OsBIERF3, and OsBIERF4 exhibit expression regulated by salt stress (Cao et al., 2006). The pink module consisted of 150 genes, including two hub genes (*Os03g0758900* and *Os01g0730700*), both of which encode WRKY transcription factors. These hub genes play critical regulatory roles in processes such as plant hormone signal transduction, metabolic pathways, peroxisome function, and biosynthesis of secondary derivatives, suggesting their potential involvement in salt stress response mechanisms.

## 4 Discussion

### 4.1 *OsMADS31* is a positive regulator of plant development and stress response in rice

MADS-box proteins are a family of transcription factors characterized by a conserved MADS-box domain of approximately 58–60 amino acids at the N-terminus, which primarily functions in DNA binding (West et al., 1997; Yanofsky et al., 1990). In plants, MADS-box transcription factors are involved in nearly all critical growth and developmental processes (Alvarez-Buylla et al., 2000; Rounsley et al., 1995). Many MADS-box genes regulate plant development by interacting with other MADS-box proteins. Studies have shown that *OsMADS29* in rice forms a heterodimer with its paralog *OsMADS31* to cooperatively regulate seed development (Theißen and Becker, 2004; Nayar et al., 2014). In Arabidopsis, *AGL24* and *SVP* participate in shoot development and floral transition by interacting with other proteins at different developmental stages (Michaels et al., 2003). A total of 61 MIKC-type MADS genes have been identified in rice, exhibiting highly conserved amino acid residues (**Supplementary Figure 1**). Conserved gene sequence and domain analyses revealed that all possess MADS and K-box structures, along with sequence signatures of 12 conserved amino acid motifs (**Supplementary Figure 2**). Comparative phylogenetic analysis indicated that the rice *OsMADS31* gene is closely related to homologs in maize (**Supplementary Figure 3**). Most MIKC-type MADS genes in plants are Type II and exhibit plant-specific features (Nam et al., 2003). The conservation and specificity of the MIKC-type MADS domain provide critical insights for further functional studies in plant development and stress responses. OsMADS31 is localized to the nucleus (**Figure 2**). RT-qPCR and GUS staining demonstrated its widespread expression across multiple rice tissues. It responds to salt stress, with expression levels under 6‰ NaCl treatment reaching 10-fold higher than baseline after 24 hours (**Figure 1**, **Supplementary Figure 4**). Knockout of *osmads31* led to significant reductions in agronomic traits, while overexpression promoted increased panicle length and grain number (**Supplementary Figure 6** & **7**). Analyses of the conserved specificity of the MIKC-type MADS gene domain and its expression patterns suggest that *OsMADS31* acts as a positive regulator of rice development and stress responses.

### 4.2 *OsMADS31* is a positive regulator of salt stress tolerance

The seed germination and seedling growth stages of rice are periods during which rice are most sensitive to external environmental factors (Zhang et al., 2014). Therefore, rice seed germination and seedling growth are often used as key indicators for evaluating salt stress tolerance (Yu et al., 2018). The MADS-box transcription factor AGL21 is a negative regulator of seed germination and post-germination growth and thus, plays a role in seed germination under abiotic stress. *AGL21*-overexpressing plants exhibit hypersensitivity to ABA, salt, and osmotic stress during seed germination and early growth, whereas *agl21* mutants show reduced sensitivity (Yu et al., 2017). In a GWAS study on salt stress during rice germination, the candidate gene *OsMADS31*, a member of the MADS-box transcription factor family, was predicted to contribute to salt tolerance during germination despite its downregulation under salt stress conditions (Yu et al., 2018). Using mRNA in identification of unannotated salinity stress-inducible transcripts in rice (*Oryza sativa* L.) demonstrated that *OsMADS31* expression was upregulated in roots after salt stress (Mizuno et al., 2010). The *osmads31* knockout reduced salt stress tolerance, inhibiting shoot and root growth during germination, while *osmads31* overexpression promoted growth (**Figure 3**). These results demonstrate that *OsMADS31* significantly stimulates rice seed germination under salt stress. Rice is highly sensitive to salinity during early seedling growth (Nam et al., 2015), making improved salt tolerance crucial for seedling establishment and yield under stress. Under salt stress at the three-leaf stage, *osmads31* knockout lines exhibited wilting and reduced survival rates, whereas *osmads31*overexpression enhanced survival (**Figure 4**). Increases in reactive oxygen species (ROS) generated by oxidative stress can damage lipids, proteins, nucleic acids, and membranes. Plants counteract salt-induced oxidative stress by deploying antioxidants such as catalase (CAT), peroxidase (POD), and superoxide dismutase (SOD) (Liu et al., 2019). In this study, *osmads31* knockout lines showed significantly lower CAT, POD, and SOD activities than the wild-type (WT), which was accompanied by reduced antioxidant enzyme activity and increased oxidative damage. By contrast, *osmads31* overexpression lines displayed elevated CAT, POD, and SOD levels (**Figure 5**), which increased antioxidant capacity and stress-responsive metabolite accumulation, facilitating ROS scavenging and mitigated membrane lipid peroxidation. These findings indicate that *OsMADS31* strengthens antioxidant defenses, thereby reducing salt stress damage.

### 4.3 Mechanism of *OsMADS31*-mediated increase in salt stress tolerance in rice

Reactive oxygen species (ROS), including H_2_O_2_, O_2_^-^, and hydroxyl radicals (•OH), are chemical compounds that can cause cellular membrane damage, lipid peroxidation, and malondialdehyde (MDA) production (D’Autreaux and Toledano, 2007). The MDA level serves as a common indicator of membrane lipid peroxidation and indirectly reflects plant stress resistance (Zhang et al., 2013). Overexpression of *SlMBP11* in tomato enhanced salt tolerance, as evidenced by lower relative electrolyte leakage and reduced MDA content in transgenic plants (Guo et al., 2016), suggesting that MADS-box genes may participate in antioxidant systems to confer salt tolerance. Following salt stress treatment, *OsMADS31*-overexpressing (*OE*) lines showed significantly lower MDA levels than those of wild-type (WT) and knockout lines (**Figure 5E**), indicating reduced oxidative damage in *OE* plants under salt stress. Previous studies have demonstrated that salt stress can increase antioxidant enzyme activity while inducing ROS production and accumulation, thereby regulating plant salt tolerance (Miller et al., 2010; Wahid et al., 2007; Yan et al., 2014). Under salt stress, *osmads31* knockout lines exhibited more intense DAB and NBT staining than the WT, while *osmads31* overexpression lines showed minimal staining (**Figure 4E, F**). Additionally, osmolyte accumulation is a crucial response to salt stress, with proline being the most extensively accumulated compound (Wang et al., 2015). Proline scavenges ROS, stabilizes subcellular structures, and regulates redox homeostasis (Szabados and Savoure, 2010). The *osmads31* knockout lines accumulated significantly less proline than the WT under salt stress, while *osmads31* overexpression lines showed the opposite trend (**Figure 5D**). Physiological studies on rice salt tolerance have identified proline as a key osmoprotectant (Ozturk et al., 2021) that safeguards cellular proteins and membrane structures from degradation or damage, thereby improving seedling salt tolerance (Li et al., 2018) In this study, *OsMADS31*-*OE* lines displayed markedly increased proline accumulation under salt stress at the three-leaf stage (**Figure 5D**), suggesting that enhanced salt tolerance in *OE* plants may correlate with proline biosynthesis. Transcriptome analysis revealed that salt stress alters specific gene expression patterns involving three regulatory networks: protein kinases, catalases, and transcription factors. KEGG enrichment analysis highlighted carbon metabolism and amino acid biosynthesis/metabolism as major pathways affected by salt stress, together with glycolysis/gluconeogenesis, lipid metabolism, and plant hormone signal transduction (**Supplementary Figure 10**). WGCNA identified differentially expressed gene modules participating in secondary metabolite biosynthesis/metabolic pathways, where hub genes played pivotal regulatory roles in plant hormone signaling, metabolic pathways, and peroxisome processes (**Figure 6**), indicating their involvement in salt stress responses. Together, the results provide evidence that *OsMADS31* improves salt tolerance by upregulating antioxidant-related genes, activating antioxidant enzymes, and reducing oxidative damage. However, the molecular mechanisms via which it exerts these effects will require further investigation, particularly with respect to how *OsMADS31*-mediated stress signaling pathways enhance crop salt tolerance.

## 5 Conclusions

In summary, this study demonstrates a novel role of the MADS-box gene *OsMADS31* in salt stress tolerance. To validate the function of OsMADS31 transcription factor in salt tolerance, we generated *osmads31* knockout (*KO*) mutants and *OsMADS31* overexpression (*OE*) lines in the Nipponbare background. The *KO* mutants showed significantly altered panicle morphology and significant reductions in seed-setting rate, panicle length, grain number per panicle, and 1000-grain weight, whereas *OE* lines exhibited the opposite trends. Under salt stress, *KO* mutants showed severely inhibited growth both during seed germination and at the three-leaf stage, whereas *OE* lines maintained normal germination and growth. After salt treatment and recovery at the three-leaf stage, *KO* mutants displayed significantly lower survival rates compared with WT plants, while *OE* lines grew normally. Physiological assays revealed that *KO* mutants exhibited significantly lower CAT, POD, and SOD activities and reduced Pro content, along with stronger DAB and NBT staining and higher MDA levels, than the WT; conversely, *OE* lines showed the opposite trends. Transcriptome analysis of *osmads31*-*KO* mutants under salt stress revealed significant enrichment of genes related to plant hormone signal transduction and biosynthesis pathways, suggesting their active involvement in salt stress tolerance. Based on the results of GO and KEGG enrichment analyses of DEGs, we propose that *OsMADS31* plays important roles in salt stress signaling pathways.

## Data Availability

The datasets presented in this study can be found in online repositories. The names of the repository/repositories and accession number(s) can be found in the article/**Supplementary Material**.
