# Supplementary material for: MIKC-type MADS-box transcription factor OsMADS31 positively regulates salinity tolerance in rice
Source: Front Plant Sci. 2025 Sep 4;16:1628305. doi: 10.3389/fpls.2025.1628305 (PMC12443732; doi:10.3389/fpls.2025.1628305)
Supplement: Supplementary file 1 [file DataSheet1.pdf]

Supplementary Material

1 Supplementary Data

Supplementary Material should be uploaded separately on submission. Please include any supplementary data, figures and/or tables.

Supplementary material is not typeset so please ensure that all information is clearly presented, the appropriate caption is included in the file and not in the manuscript, and that the style conforms to the rest of the article.

2 Supplementary Figures and Tables

For more information on Supplementary Material and for details on the different file types accepted, please see here.

2.1 Supplementary Figures

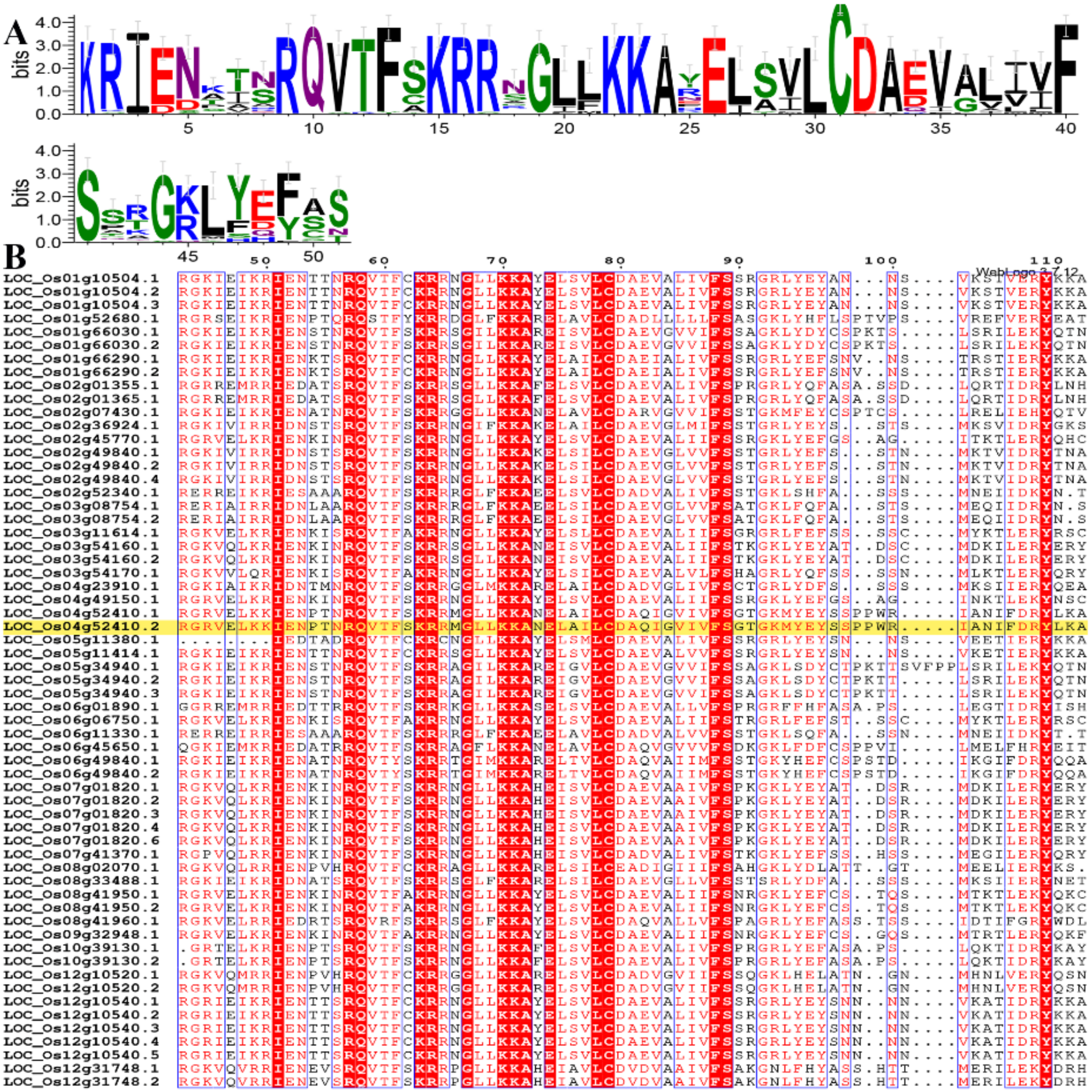

Supplementary Figure 1. Conservation and amino acid sequence analysis of MIKC-type MADS domains in rice. (A) Analysis of DNA binding conserved domains, sequence logos of conserved domains were generated using WebLogo 3 (https://weblogo.threeplusone.com/); (B) amino acid sequence alignment of DNA binding conserved domains, the red-shaded parts are conservative points, marked yellow is OsMADS31.

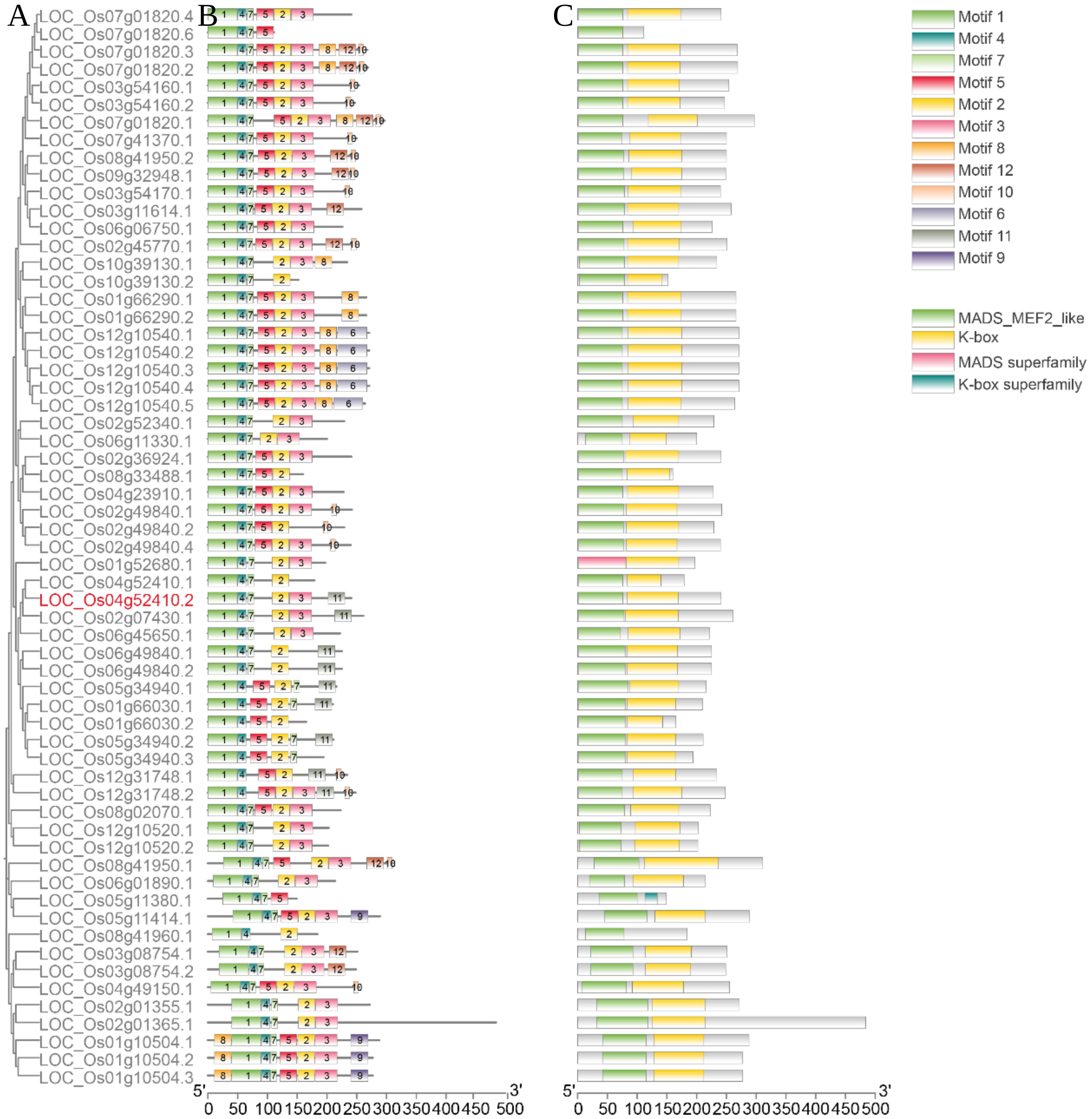
A
B
C
LOC_Os07g01820.4
LOC_Os07g01820.6
LOC_Os07g01820.3
LOC_Os07g01820.2
LOC_Os03g54160.1
LOC_Os03g54160.2
LOC_Os07g01820.1
LOC_Os07g41370.1
LOC_Os08g41950.2
LOC_Os09g32948.1
LOC_Os03g54170.1
LOC_Os03g11614.1
LOC_Os06g06750.1
LOC_Os02g45770.1
LOC_Os10g39130.1
LOC_Os10g39130.2
LOC_Os01g66290.1
LOC_Os01g66290.2
LOC_Os12g10540.1
LOC_Os12g10540.2
LOC_Os12g10540.3
LOC_Os12g10540.4
LOC_Os12g10540.5
LOC_Os02g52340.1
LOC_Os06g11330.1
LOC_Os02g36924.1
LOC_Os08g33488.1
LOC_Os04g23910.1
LOC_Os02g49840.1
LOC_Os02g49840.2
LOC_Os02g49840.4
LOC_Os01g52680.1
LOC_Os04g52410.1
LOC_Os04g52410.2
LOC_Os02g07430.1
LOC_Os06g45650.1
LOC_Os06g49840.1
LOC_Os06g49840.2
LOC_Os05g34940.1
LOC_Os01g66030.1
LOC_Os01g66030.2
LOC_Os05g34940.2
LOC_Os05g34940.3
LOC_Os12g31748.1
LOC_Os12g31748.2
LOC_Os08g02070.1
LOC_Os12g10520.1
LOC_Os12g10520.2
LOC_Os08g41950.1
LOC_Os06g01890.1
LOC_Os05g11380.1
LOC_Os05g11414.1
LOC_Os08g41960.1
LOC_Os03g08754.1
LOC_Os03g08754.2
LOC_Os04g49150.1
LOC_Os02g01355.1
LOC_Os02g01365.1
LOC_Os01g10504.1
LOC_Os01g10504.2
LOC_Os01g10504.3
5'
3'
0 50 100 150 200 250 300 350 400 450 500
Motif 1
Motif 4
Motif 7
Motif 5
Motif 2
Motif 3
Motif 8
Motif 12
Motif 10
Motif 6
Motif 11
Motif 9
MADS_MEF2_like
K-box
MADS superfamily
K-box superfamily

D

Number	Logo	E-value	Sites	Width
1.	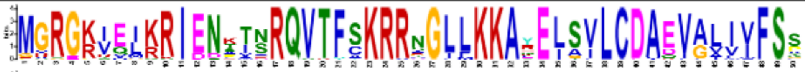	1.6e-2429	61	50
2.	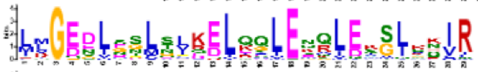	8.6e-578	55	29
3.	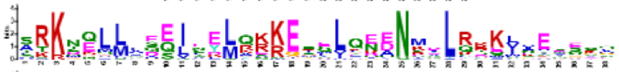	5.0e-378	42	38
4.	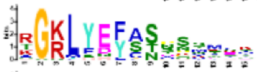	1.3e-326	61	15
5.	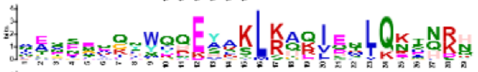	1.1e-340	42	29
6.	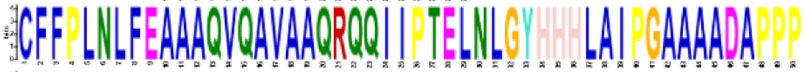	1.6e-148	5	50
7.	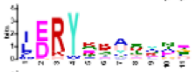	5.9e-131	61	11
8.	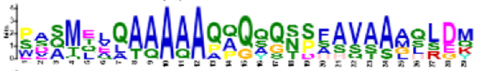	3.5e-092	13	29
9.	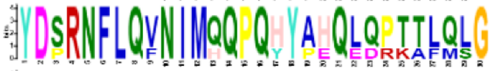	3.2e-039	4	30
10.	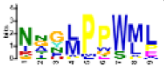	8.0e-037	17	9
11.	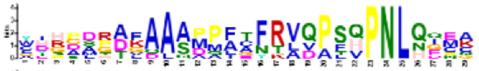	5.5e-050	9	29
12.	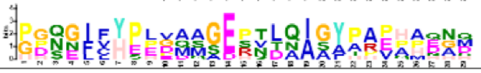	4.5e-028	8	29

Supplementary Figure 2. Conserved motifs and domains of MIKC-type MADS-box proteins in rice. (A-D) Phylogenetic tree (A), motif composition (B), domain architecture of MIKC-type MADS-box proteins in rice (C), and motif 1-12 (D). The phylogenetic tree was constructed using MEGA 7.0 with the neighbor-joining method, based on the amino acid sequences of MIKC-type MADS-box proteins found in rice. Conserved motifs and domains of MIKC-type MADS-box proteins were analyzed using the MEME 5.1.7 tool. Structural annotations of all MIKC-type MADS-box genes were obtained using TBtools-II v2.142.

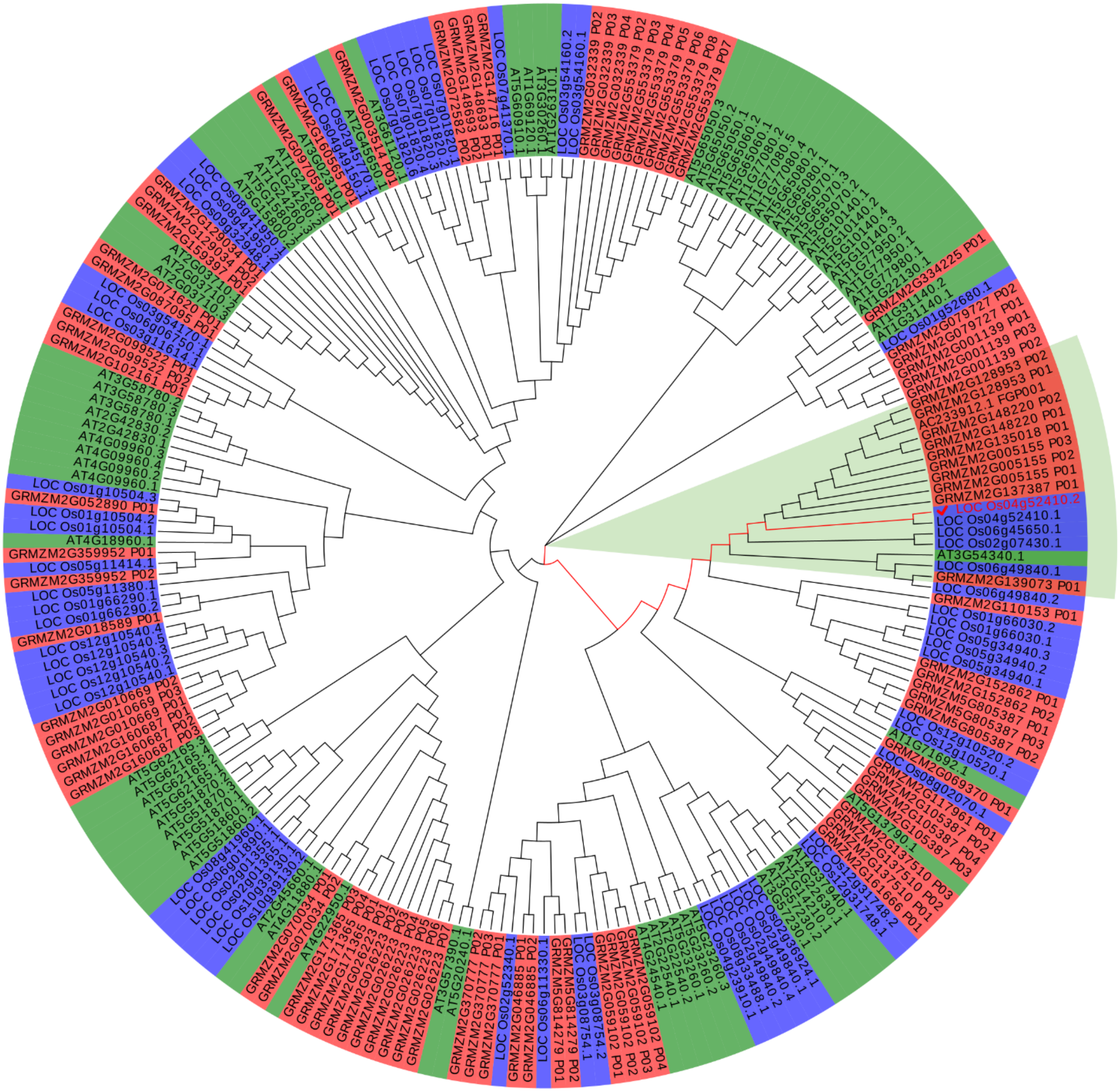

Supplementary Figure 3. Phylogenetic analysis of MIKC-type MADS-box genes. Phylogenetic tree of the amino acid sequences of MIKC-type MADS-box transcription factors found in rice (*Oryza sativa* L), Arabidopsis (*Arabidopsis thaliana*), and maize (*Zea mays*) was constructed using the neighbor-joining method in MEGA 7.0. The tree was annotated using the online tool Evolview. MADS-box transcription factors of rice, Arabidopsis, and maize are highlighted in blue, green, and red, respectively. The red branch and shaded area indicate the clade closely related to the *OsMADS31* gene.

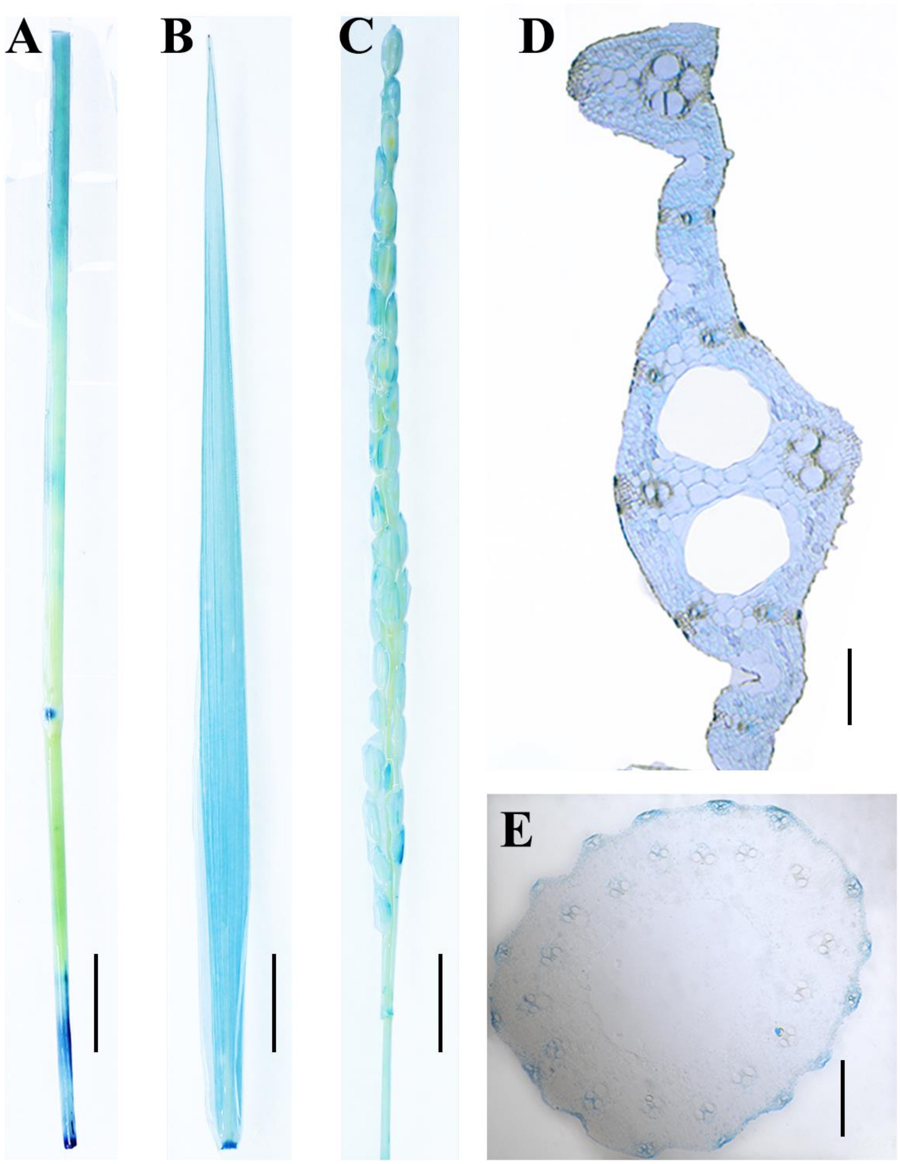

Supplementary Figure 4. GUS staining assays. (A-E) GUS staining of *pOsMADS31::GUS* transgenic rice plants. Images of stem (A), leaf (B), young panicle (C), and the cross-sections of leaf (D) and stem (E) are shown. Data represent the mean ± SD of three independent replicates. Scale bars: 5 cm (A-C), 20 μm (D-E).

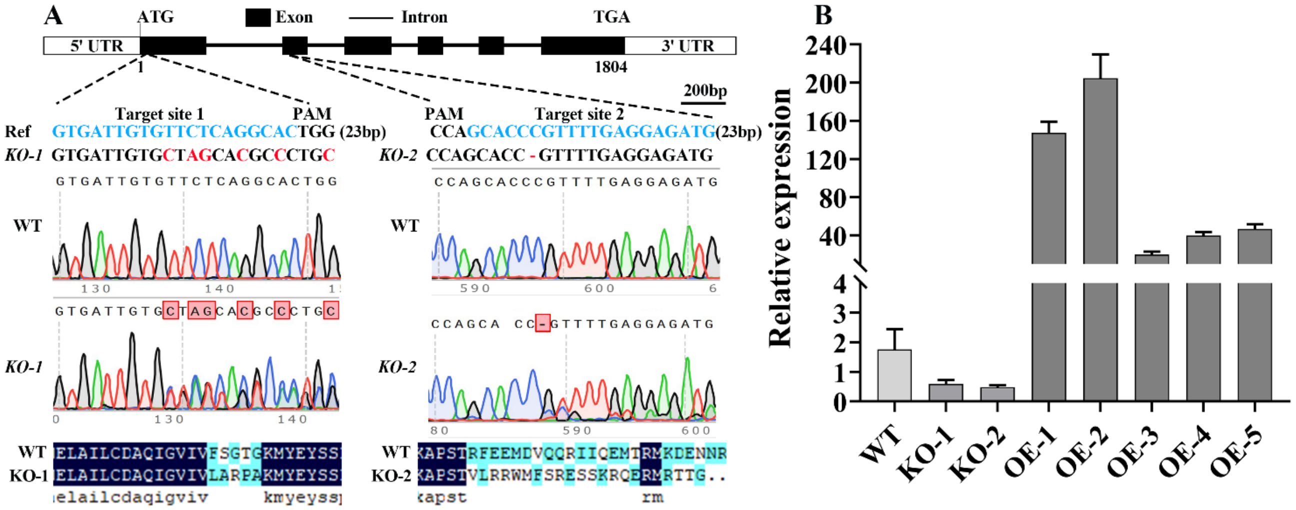

Supplementary Figure 5. (A) Schematic representation of the *OsMADS31* gene structure and of target sites selected for generating *osmads31* knockout mutants using the CRISPR-Cas9 technology. *OsMADS31* nucleotide sequence and its deduced amino acid sequence are compared between wild-type (WT) and knockout mutant lines (*KO-1* and *KO-2*). (B) Expression analysis of *OsMADS31* in the knockout mutants and overexpression lines. Expression analysis of *OsMADS31* in WT plants, *osmads31* knockout mutants (*KO-1* and *KO-2*), and *OsMADS31* overexpression lines (*OE-1* to *OE-5*) by RT-qPCR. Data represent the mean ± SD of three independent replicates.

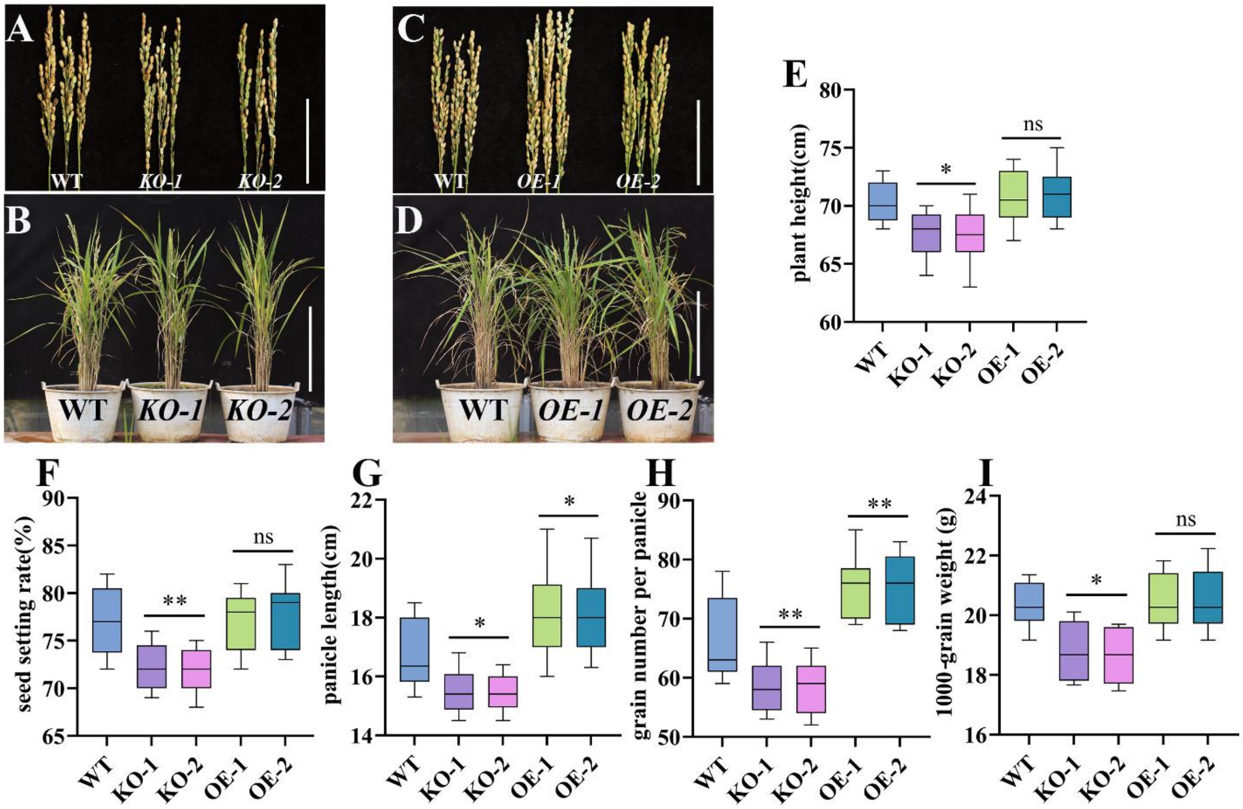

Supplementary Figure 6. Phenotypic and agronomic trait analyses of *osmads31* knockout mutants (*KO-1* and *KO-2*), *OsMADS31* overexpression lines (*OE-1* and *OE-2*), and wild-type (WT) plants at maturity. (A-D) Photographs showing the single panicle (A) and whole plant (B) phenotypes of WT and *KO* plants at maturity as well as the single panicle (C), and whole plant (D) of WT and *OE* plants. Scale bars = 5 cm. (E-I) Quantitative analysis of the plant height (E), seed-setting rate (F), panicle length (G), grain number per panicle (H), and 1000-grain weight (I) in WT, *KO*, and *OE* plants. Data represent the mean ± SD of three independent replicates. Asterisks indicate statistically significant differences compared with the WT (**P* < 0.05, ***P* < 0.01). ns, no significant difference.

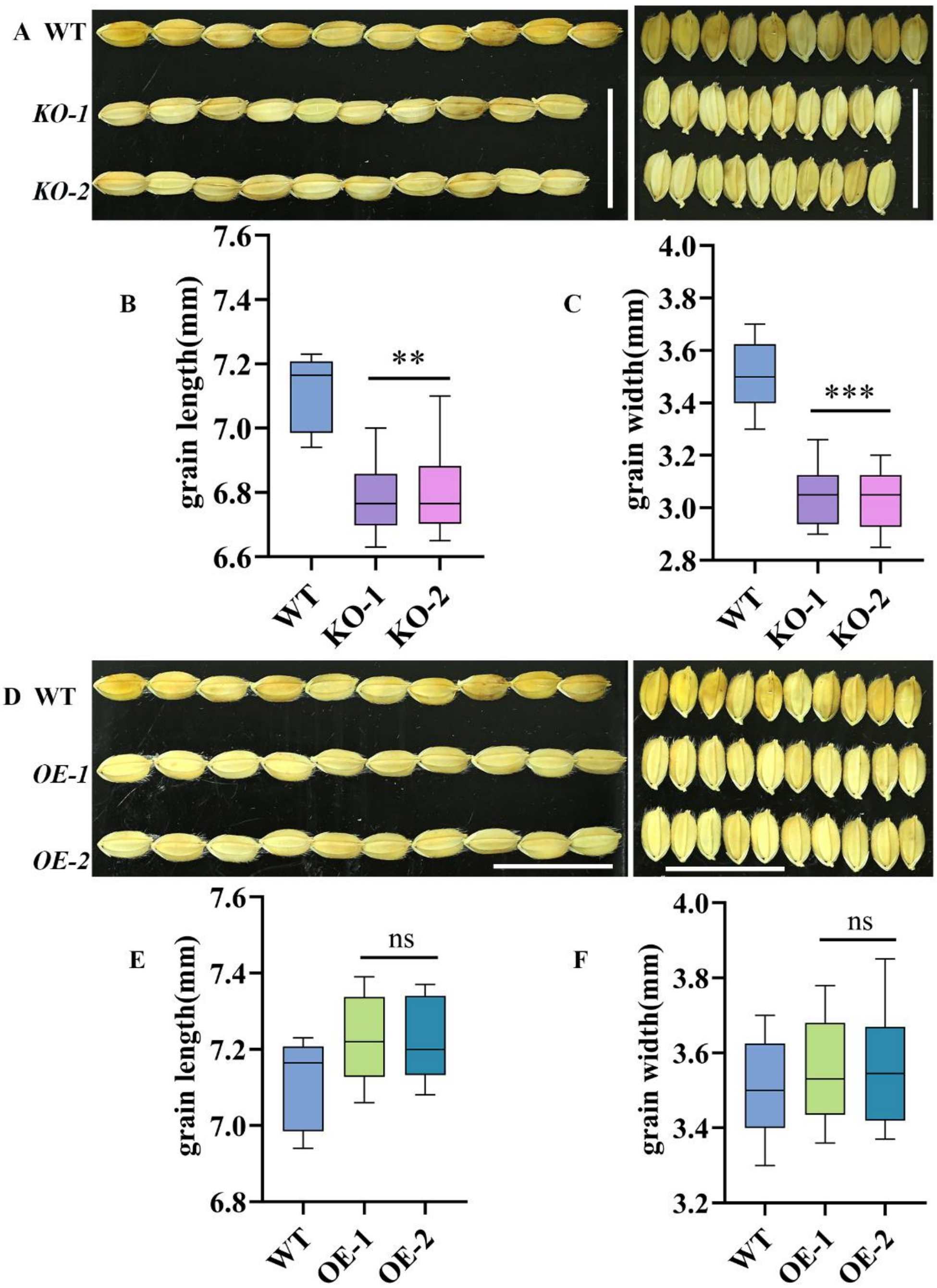

Supplementary Figure 7. Analyses of *osmads31* knockout mutants (*KO-1* and *KO-2*), *OsMADS31* overexpression lines (*OE-1* and *OE-2*), and wild-type (WT) plants at grain length and width. (A-F) Photographs showing the grain phenotypes (A), grain length (B) and grain width (C) of WT and *KO* plants at maturity as well as the grain phenotypes (D), grain length (E) and grain width (F) of WT and *OE* plants. Scale bars = 5 cm. Data represent the mean ± SD of three independent replicates. Asterisks indicate statistically significant differences compared with the WT (***P* < 0.01, ****P* < 0.001). ns, no significant difference.

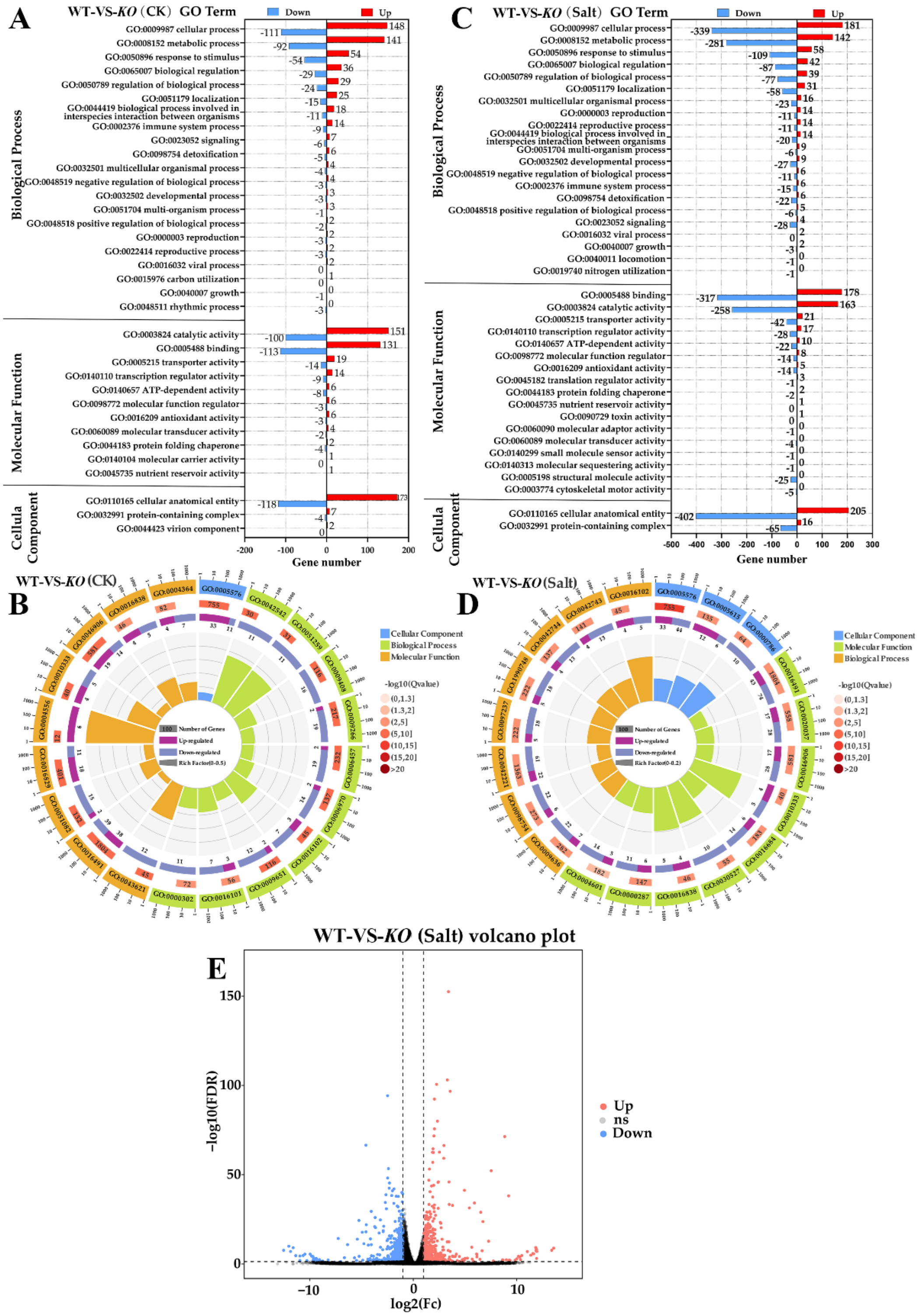

Supplementary Figure 8. GO enrichment analysis of DEGs. (A, C) Secondary bar plots of differential GO enrichment before (A) and after (C) the salt stress treatment. (B, D) GO enrichment circle plots pre-treatment (B) and post-treatment (D). (E) Volcano plot of differential genes after the salt stress treatment.

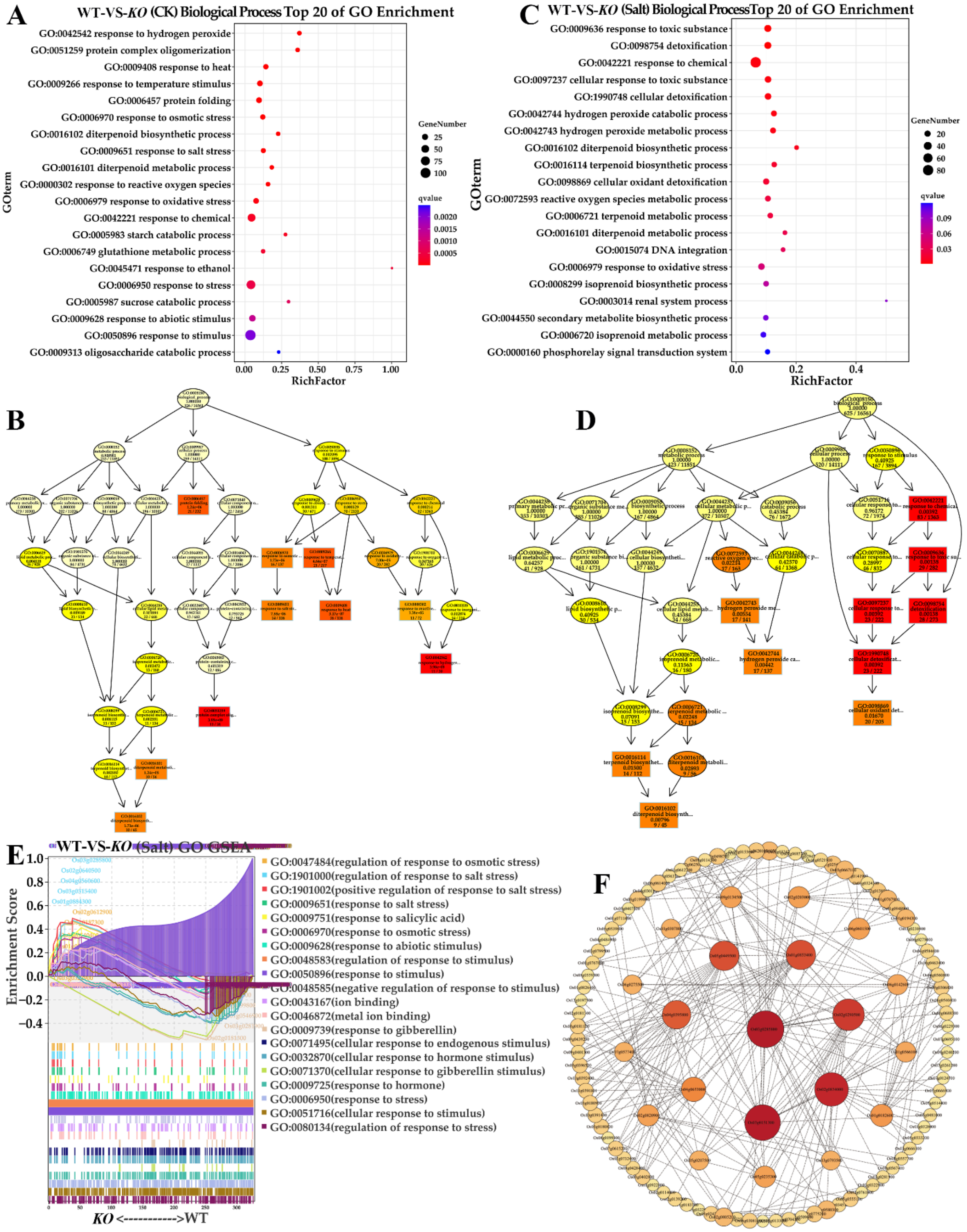

Supplementary Figure 9. GO enrichment analysis of DEGs. (A) Pre-treatment GO enrichment bubble plot; (B) Pre-treatment GO directed acyclic graph (DAG); (C) Post-salt-stress GO enrichment bubble plot; (D) Post-treatment GO DAG; (E) Gene Set Enrichment Analysis (GSEA) of ‘response to stimulus’; (F) Regulatory network diagram.

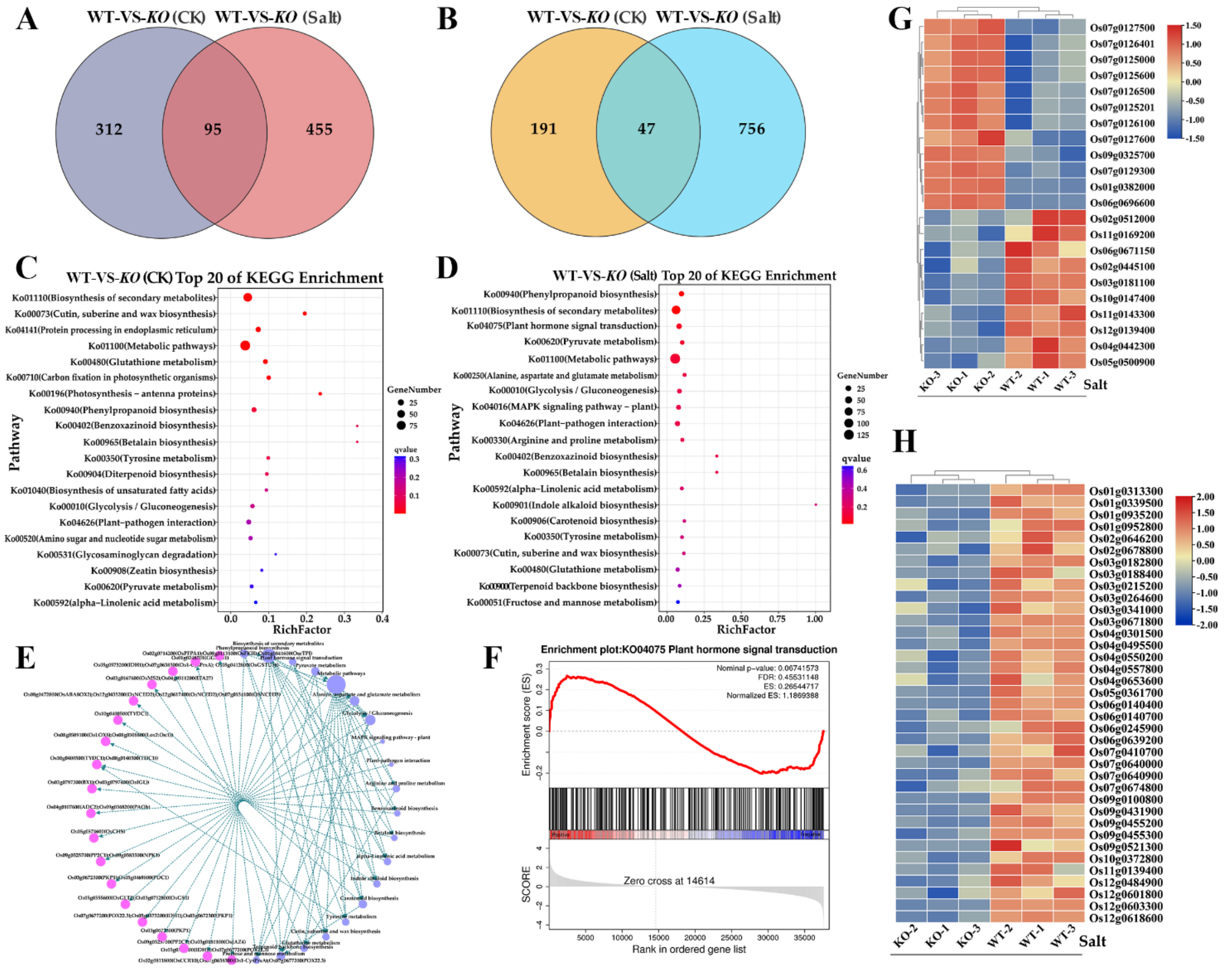

Supplementary Figure 10. KEGG enrichment analysis of DEGs. (A, B) Venn diagrams showing the numbers of genes differentially upregulated (A) and downregulated (B) in *KO* mutants relative to the WT under control and salt stress conditions. (C, D) KEGG enrichment analysis of DEGs identified in the WT-vs-*KO* comparison pre-treatment (C) and post-treatment (D). (E) KEGG hierarchy map of the top 20 enriched pathways in WT-vs-*KO* post-treatment. (F) GSEA of plant hormone signal transduction; (G) Heatmap of genes involved in plant hormone signal transduction. (H) Downregulated transcription factor-encoding genes after salt stress treatment.

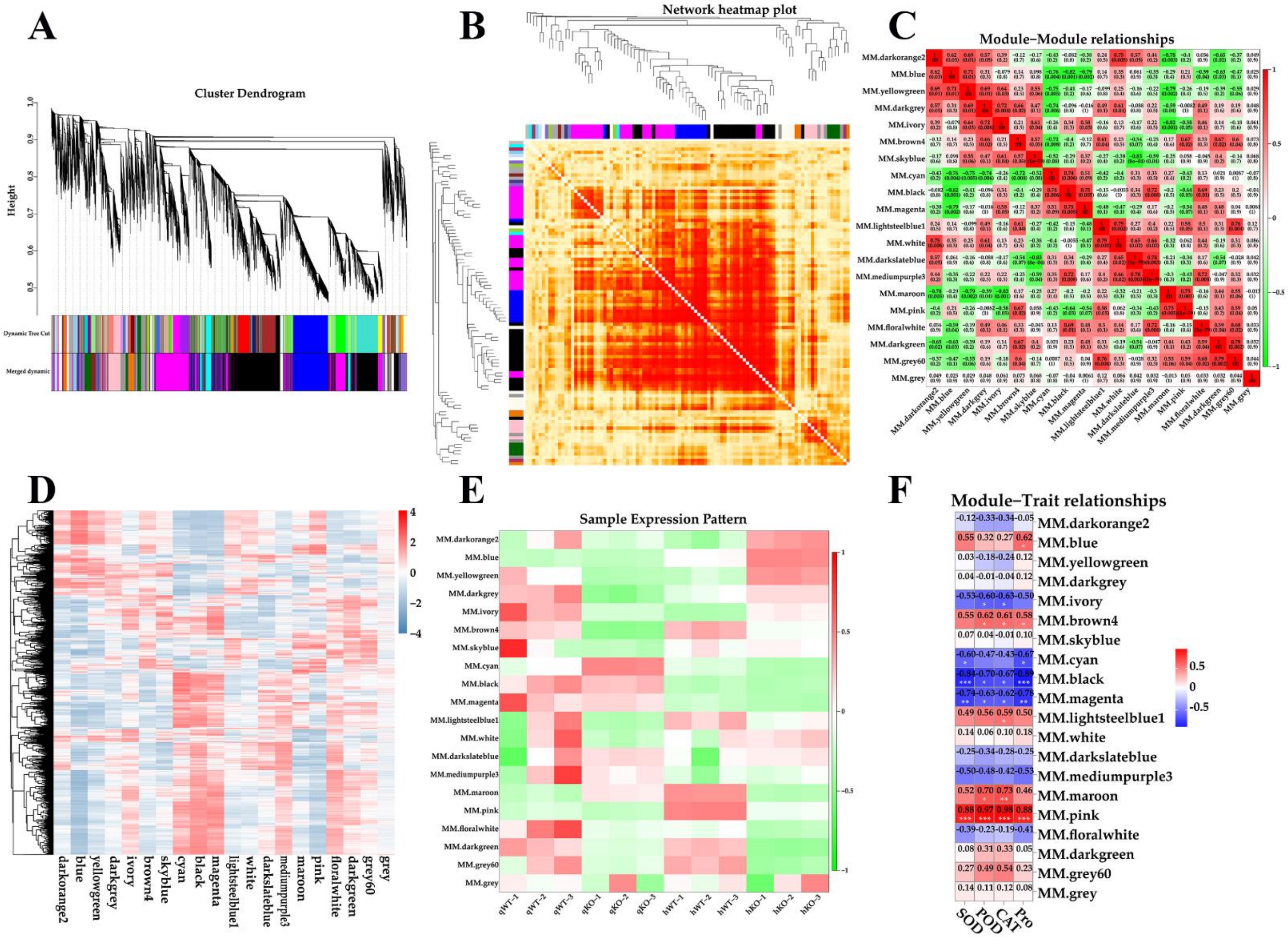

Supplementary Figure 11. Co-expression analysis of *OsMADS31*-associated genes. (A) WGCNA module dendrogram. (B-E) Heatmaps showing gene correlations within modules (B), inter-module correlations (C), gene expression patterns across modules (D), and sample expression patterns (E). (F) Module-trait association plot.

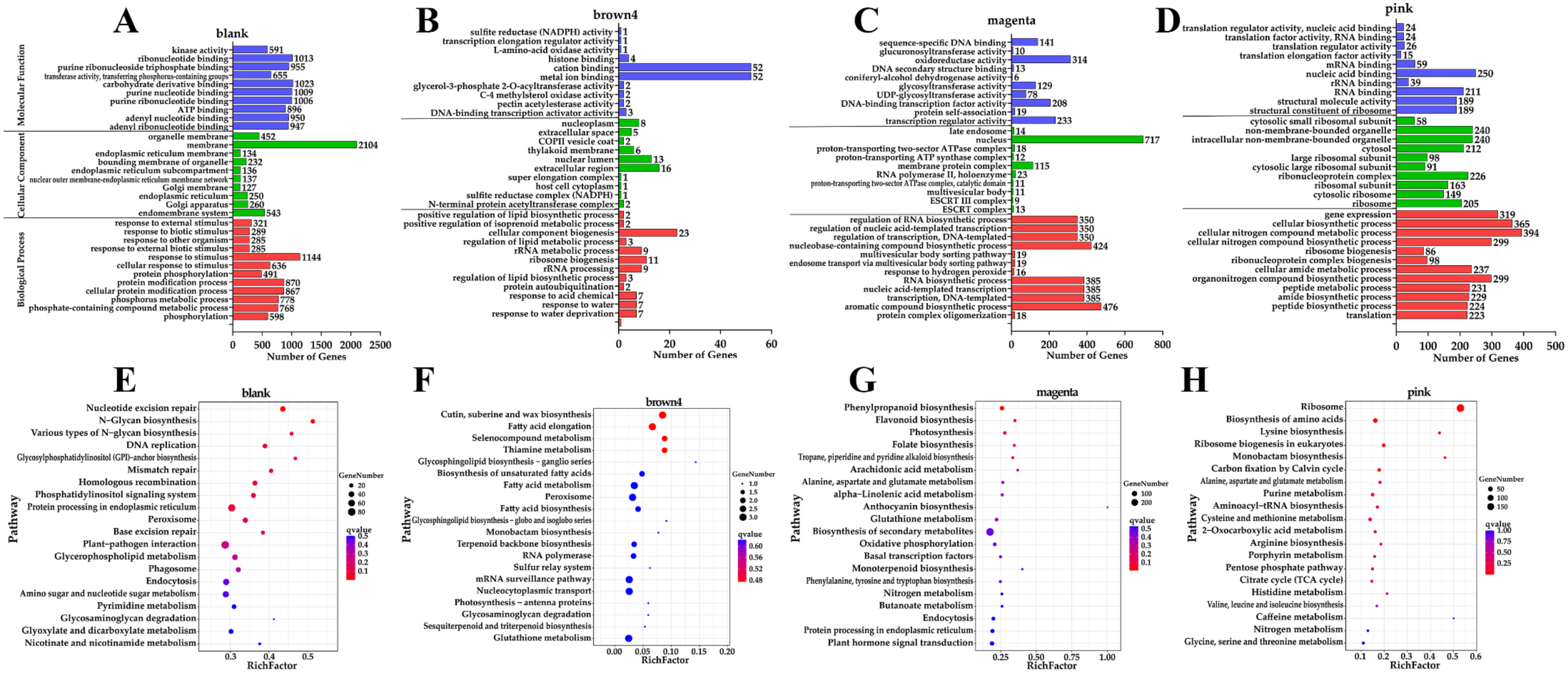

Supplementary Figure 12. Enrichment analysis of five DEGs in the modules showing significant associations. (A-D) Results of GO enrichment analysis of DEGs in the black module (A), brown4 module (B), magenta module (C), and pink module (D). (E-H) Top 20 pathways identified via the KEGG enrichment analysis of genes in the black module (E), brown4 module (F), magenta module (G), and pink module (H).

frontiers
